# Lattice structure musculoskeletal robots: Harnessing programmable geometric topology and anisotropy

**DOI:** 10.1126/sciadv.adu9856

**Published:** 2025-07-16

**Authors:** Qinghua Guan, Benhui Dai, Hung Hon Cheng, Josie Hughes

**Affiliations:** CREATE Lab, Institute of Mechanical Engineering, School of Engineering, EPFL, Lausanne 1015, Switzerland.

## Abstract

Natural musculoskeletal systems combine soft tissues and rigid structures to achieve diverse mechanical behaviors that are both adaptive and precise. Inspired by these systems, we propose a programming method for designing bioinspired soft-rigid robotic structures using lattice geometries made from a single material. By introducing previously unknown approaches to the geometric design of unit cells within lattice structures—based on continuous blending and superposition of existing lattice geometries—we can precisely tune stiffness and anisotropy. These designs enable the creation of three-dimensional structures with spatially varying mechanical properties, ranging from tissue-like compliance to rigid, bone-like load-bearing capabilities. Using these methods, we fabricated a musculoskeletal-inspired tendon-driven robotic elephant that integrates joints with programmable bending profiles, achieving a continuously soft trunk. Our lattice geometry generation techniques allow for over 1 million discrete configurations and infinite geometric variations, offering a scalable solution for designing lightweight, adaptable robots.

## Teaser

Single-material printed tendon-driven lattice musculoskeletal robot features a continuous soft trunk and rigid bone-like profiles.

## INTRODUCTION

**Fig. 1.**
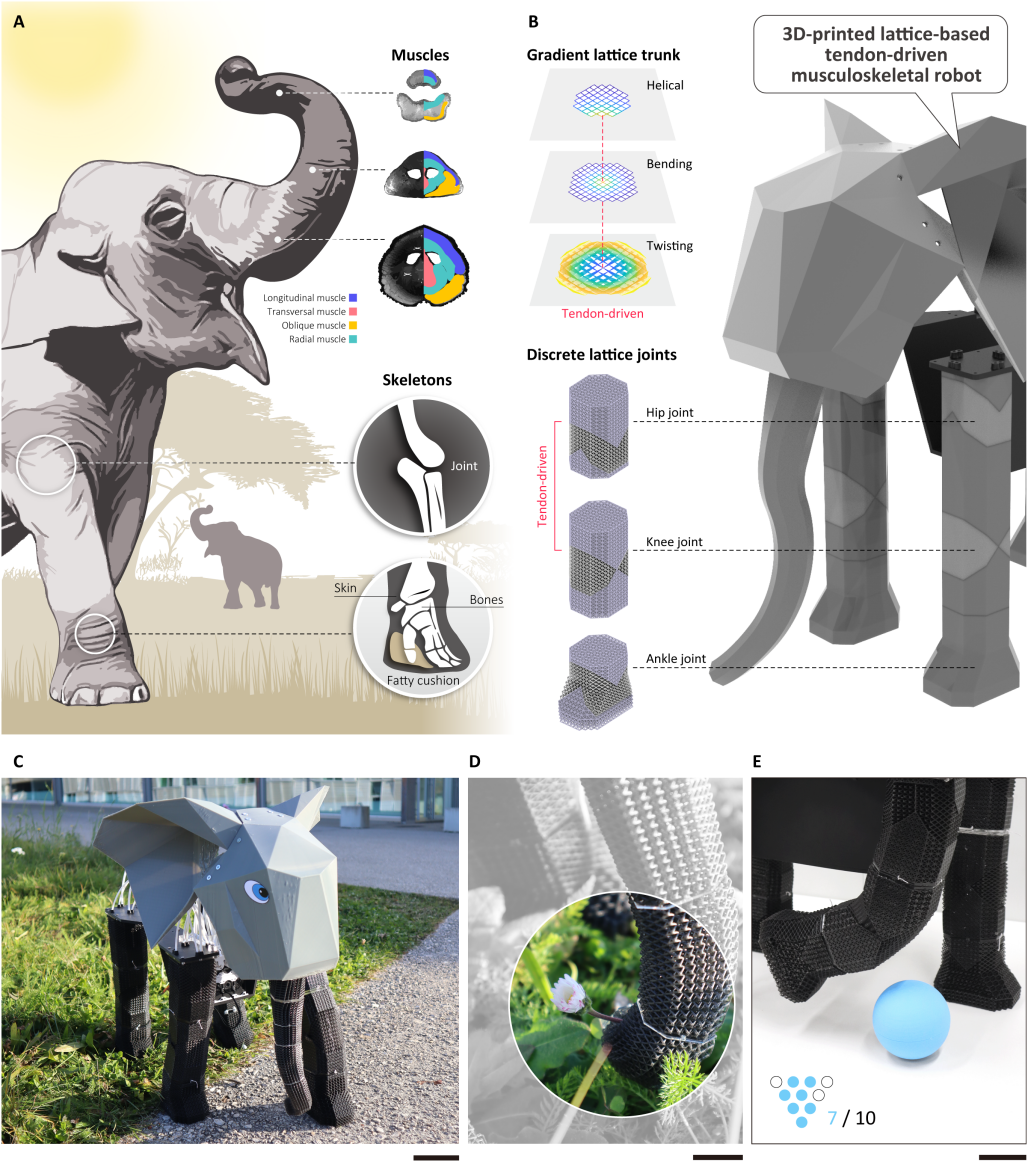
Concept of a lattice musculoskeletal robot. (**A**) Inspiration of the elephant musculoskeletal system (*47*) (the image is created by Adobe Photoshop and Adobe Illustrator). (**B**) Structure design of a 3D-printed lattice-based musculoskeletal robot. (**C**) Optical image showcasing the physical appearance of the elephant robot. (**D**) Enlarged image demonstrating a flower is pinched by the robotic trunk. (**E**) The robot is lifting its leg to kick the bowling ball. Scale bars, 10 cm (C), 2 cm (D), and 5 cm (E).

Evolutionary pressures, such as the transition from water to land (*1*) and the need for specialization within ecological niches (*2*), have resulted in animals developing complex and diverse musculoskeletal forms. The integration of softer tissues with rigid structures, some passive and others functional, provides advantages in terms of the energetics, precision, and range of movement, which enables a wide range of complex behaviors (*3*). For instance, cheetahs have elastic tendons that work alongside rigid bones to store and release energy for explosive acceleration (*4*). The arrangement and interplay of muscles, tendons, and ligaments in the human hand allows for fine motor skills (*5*), whereas the flexible spines of snakes enable flexibility of movement (*6*). These are enviable properties for robots and have inspired the development of robotic musculoskeletal lower limbs (*7*), upper bodies, and even humanoid robots (*8*). More recently, 3D printing has emerged as a key fabrication technology for creating soft-rigid musculoskeletal robots (*9*). Such multimaterial additive manufacturing techniques can enable complex three-dimensional (3D) geometries to be formed with spatially varying material properties and exhibit multimodal deformation under actuation (*10*–*12*). However, the complexity and variety of musculoskeletal structures in animals are notable, with many different joint arrangements and even fully muscular organs existing (such as the elephant trunk in Fig. 1A). Thus, we need design methods and 3D printing technologies that can imitate this wide range of underlying physiologies.

Multimaterial 3D printing has been a key technique for enabling single-stage fabrication of the structure of musculoskeletal rigid-soft systems. Notable examples include a skeleton hand enabled by two material fabrications of rigid bones and soft ligaments (*13*), vision-controlled printing (*14*), and multimaterial multinozzle 3D printing (*15*). Although multimaterial 3D printing inherently enables the combination of materials or different properties, this approach is also somewhat limited in the achievable complexity and variety of musculoskeletal structures (*16*, *17*). First, it limits the range of stiffnesses of different components of the structure to a discrete or typically small range, limiting the capacity to replicate the wide-ranging stiffnesses in a continuous variation as seen in nature. Second, the anisotropy of the material is largely fixed by its composition or printing technology, which makes it challenging to mimic the underlying anisotropy and directionality seen in musculoskeletal systems (*18*).

The development of lattice metamaterials with programmable bulk behaviors enables a more continuous and extensive range of mechanical properties from a single base material (*19*). Lattice foam structures are typically formed using repeated unit cells such as body-centered cubic (bcc) (*20*), Kelvin (*21*), or octahedron (*22*). Although these structures provide enhanced mechanical properties, their reliance on repetitive geometries constrains the range of achievable stiffness and anisotropy. Recent efforts have introduced more complex geometries, such as topological regulation or thickness modulation, to generate gradient properties (*23*–*27*). However, these approaches primarily focus on isotropic properties, thickness variation, or a restricted range of structural variants, limiting their applicability in designing highly tunable mechanical behaviors. In addition, lattice structures developed from soft materials (*28*–*30*) exhibit complex mechanical behaviors during large deformations due to material nonlinearity (e.g., superelasticity and beam buckling) during large deformation (*31*, *32*), which makes the design and optimization of these challenging. This body of work demonstrates the potential of varying the geometry of lattice structures to achieve a range of structural properties, such as programmable deformation, stiffness, Poisson’s ratio, and isotropy (*24*, *33*–*37*). However, the range of stiffness and the ability to program their anisotropy is currently limited by the range of different geometries that can be created (*38*).

In this work, we present a methodology for designing and fabricating programmable soft-rigid lattice structures using a single material. At the core of this approach is topology regulation (TR), which expands the design space of lattice unit cells by introducing a generalized lattice blending approach. This enables the creation of infinite new lattice geometries with markedly increased range of stiffness and anisotropy properties, surpassing the constraints of conventional lattice designs. By leveraging TR, we can create lattice structures with a more diverse set of mechanical behaviors, ranging from highly flexible to rigid, allowing for finer control over material properties. Building on this foundation, we integrate superposition programming (SP) with TR, which enables the spatial variation of lattice stiffness, anisotropy, and connectivity, either continuously or discretely, on the millimeter scale. The topology blending and superposition distribution govern the spatial properties of the lattice and can allow structures to gradually or abruptly transition between mechanical states. This enables blending between different mechanical states to create soft, continuum body structures and discrete transitions between mechanical properties, which can be used to design more rigid, jointed structures. To leverage these new lattice structures and blending approaches, we introduce a computational framework for motion behavior programming that can be used for both continuous regulation and discrete distribution of lattice structures. By optimizing the topology index field and lattice cell superposition, our method enables the encoding of motion behaviors directly into the structural design. This framework allows for the structural programming of deformation profiles, load-bearing capabilities, and joint behavior, exploiting structure for intelligence behaviors. To demonstrate this approach, we fabricate a musculoskeletal-inspired elephant robot that integrates both soft and rigid components printed with a single material (Fig. 1, B and C). Soft-rigid structures consist of the musculoskeletal robotic system, in which the rigid structure acts as the load-bearing skeleton, whereas the soft structures and the tendons are used as muscles to actuate various motions. The soft, muscular trunk (Fig. 1D) exhibits twisting, bending, and helical motion, whereas the rigid skeletal joints (Fig. 1E) replicate the stiffness and load-bearing properties of an elephant’s hip, knee, and foot joints. These results highlight how our structural programming framework can synthesize complex musculoskeletal properties and behaviors, bridging soft and rigid mechanics within a unified material system.

## RESULTS

**Fig. 2.**
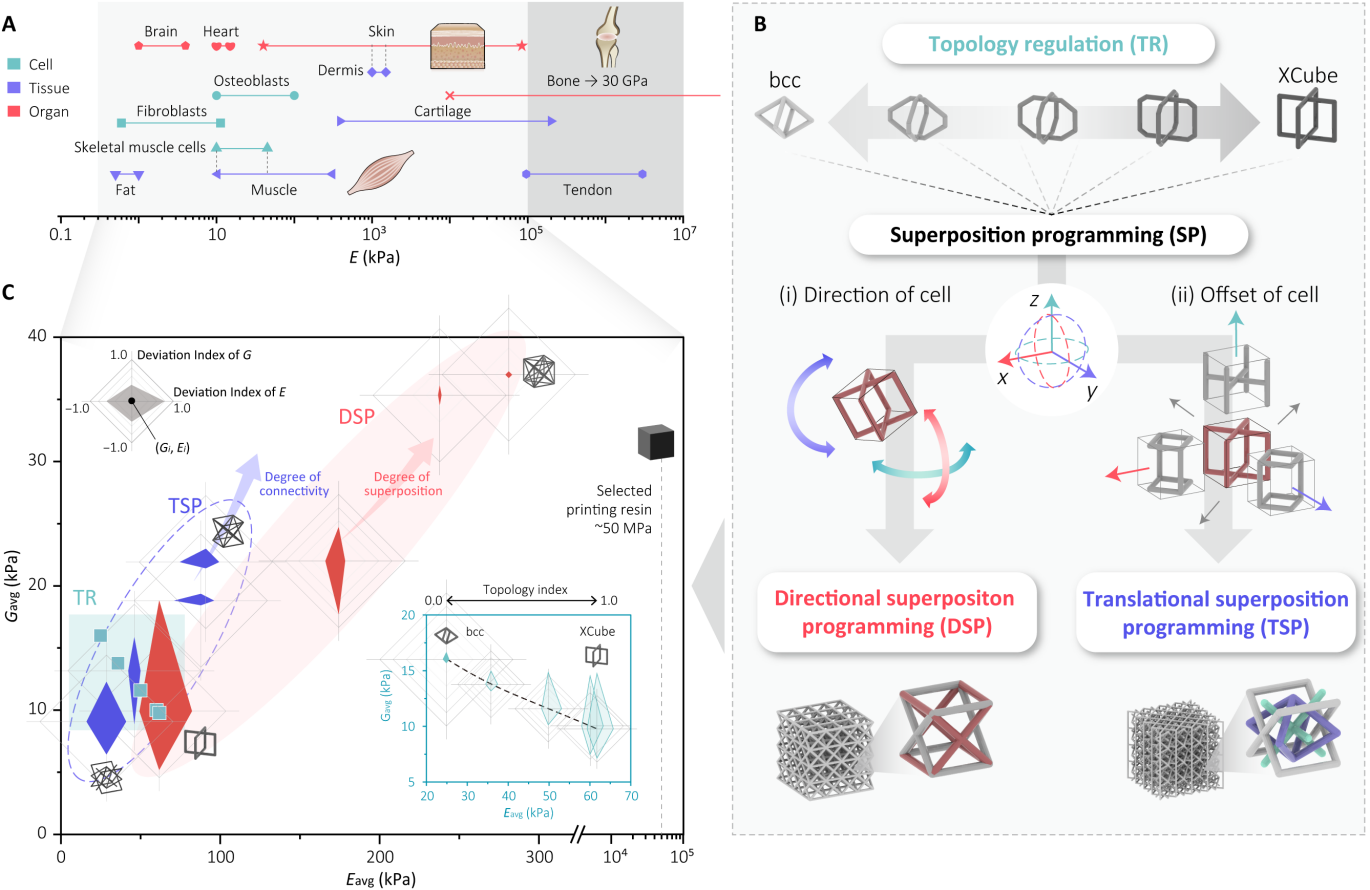
Range of mechanical properties achievable through different forms of topology programming. (**A**) Range of different Young’s moduli of tissues and structures seen in biological systems (*39*–*41*). (**B**) Two methods of geometrical programming, TR and SP. SP includes DSP and TSP. (**C**) Range of Young’s modulus and shear modulus achievable with different geometrical programming. The range of anisotropy in the shear and Young’s moduli is highlighted through the diamond-based shapes.

Human and animal musculoskeletal systems rely on a wide range of structures and tissues with stiffness values ranging from rigid bones (Fig. 2A), which can exceed 30 GPa, to soft tissues such as fat or muscle, with stiffness that can be lower than 1 kPa (*39*–*41*). The stiffness properties of these tissues are also directionally dependent, meaning they exhibit different mechanical behaviors depending on the orientation of the applied force. This anisotropy determines how forces are absorbed, generated, and applied. For example, bones have higher load-bearing capacity in specific directions, allowing for efficient compressive load transfer to the ground during motion, whereas tendons and ligaments are stronger along their length, enabling effective tensile force transmission for both flexible movements and joint stability.

**Fig. 3.**
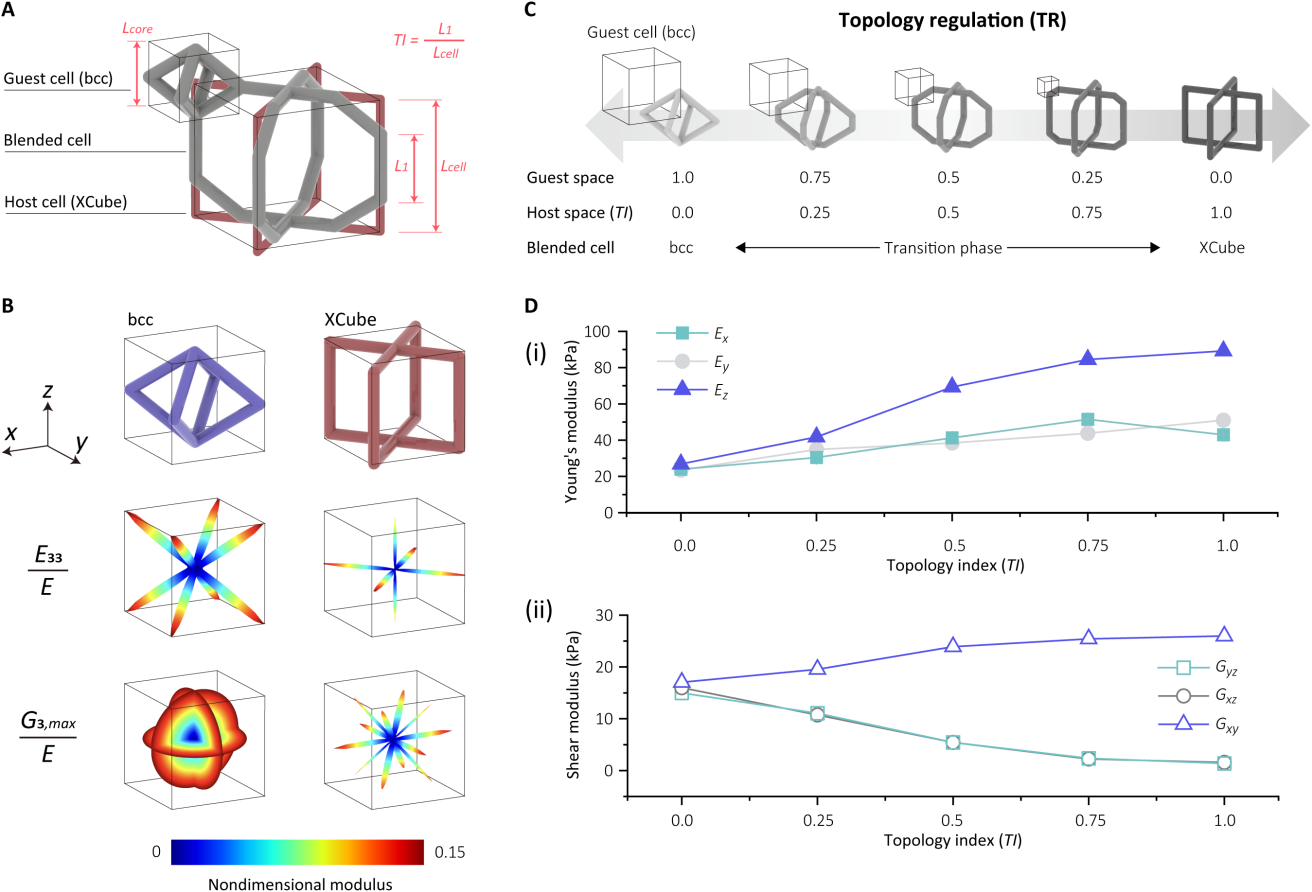
TR method for continuous blending from one geometry to another. (**A**) Schematic showing the BCB lattice structure. (**B**) Two different lattice types that we blend between bcc and XCube and their anisotropy in a nondimensional Young’s modulus and shear modulus. (**C**) Schematic showing the topology transmission between bcc and XCube. (**D**) Representation of the geometrical regulation from bcc to XCube is in a continuous manner with an increasing *TI* index.

**Fig. 4.**
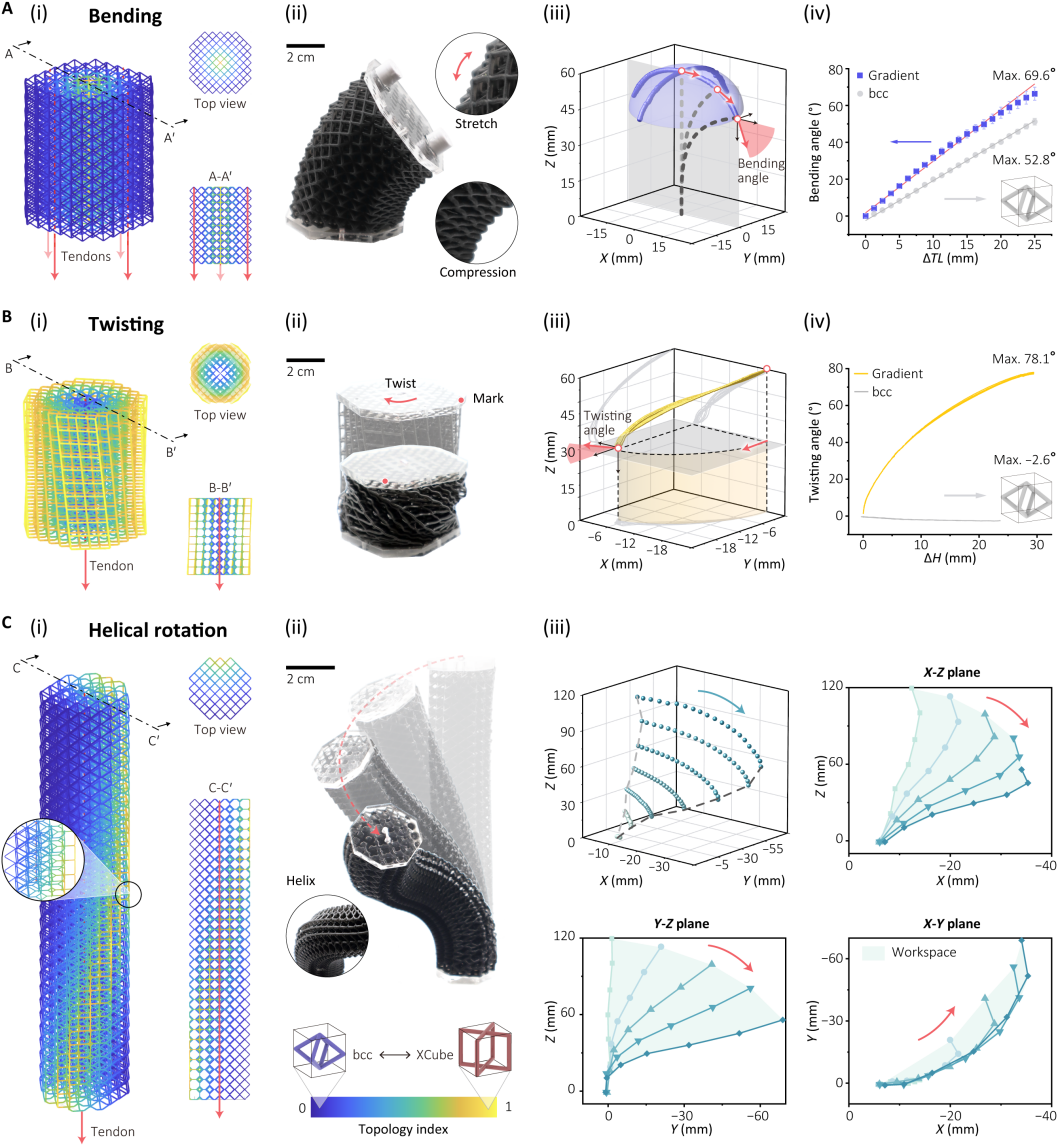
Topology-regulated muscular structures for multiple movements. (**A**) Bending movement. (**B**) Twisting movement. (**C**) Helical movement. (i) Schematics of muscular structures and tendon layout. (ii) Experimental deformation of movements. (iii) Workspace of muscular structures. (A, iv) Bending angles of muscular structures with varied tendon length (Δ*TL*). (B, iv) Twisting angles of muscular structures with varied height (Δ*H*).

**Fig. 5.**
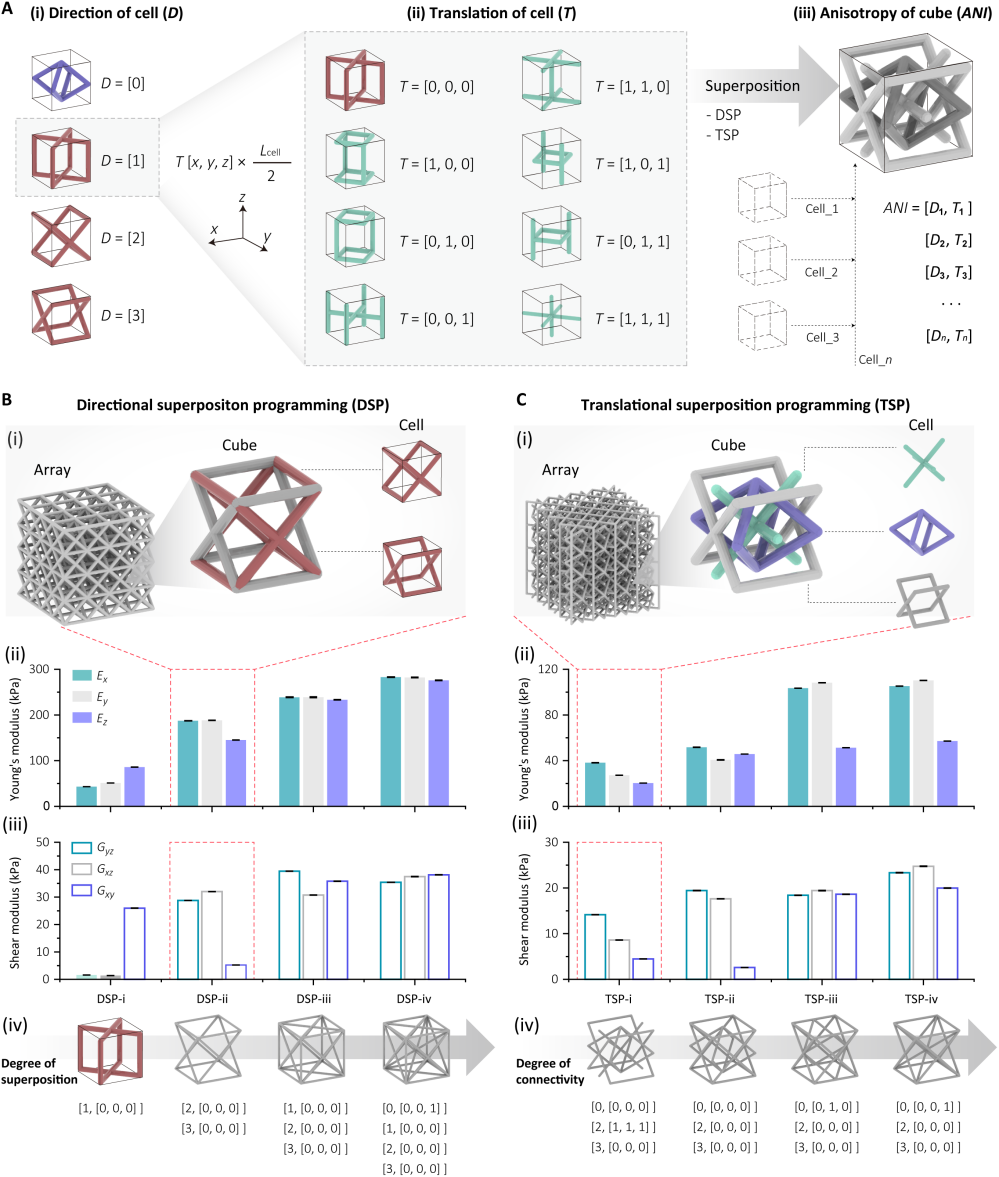
SP method for anisotropy regulation. (**A**) Two superposition methods, including changing the direction (i) and translation (ii) of a cell, can be used to program the anisotropy of a cube (iii). (**B** and **C**) Mechanical properties of cubes programmed by DSP (B) and TSP (C). (i) Enlarged structure of one specific cube sample. (ii) Young’s modulus of cubes. (iii) Shear modulus of cubes. (iv) Schematic showing the cubes with increasing superposition/connectivity.

To fabricate structures with a range of stiffness and anisotropy, we use lattice structures with spatially varying geometry. These structures can be combined with tendon-driven actuation to achieve a wide range of deformation behaviors that replicate the kinematics of musculoskeletal systems. To geometrically program these structures, we introduce two methods: topology regulation (TR) and superposition programming (SP) (Fig. 2B). The TR method allows for the continuous transition between two lattice unit cell types. This approach enables the continuous spatial blending of stiffness profiles and allows for an infinite range of blended unit cells (Fig. 3). It is particularly suited for replicating the structure of muscular organs, such as an elephant trunk (Fig. 4), where stiffness profiles vary continuously across different cross-sections. SP, the second method, superimposes different unit cells, each with a defined translation or rotation, to create new unit cells. Although this method results in discrete unit cells, it allows for a wide range of possible stiffnesses and anisotropy (Fig. 5).

Figure 2C shows the range of stiffness and anisotropy that can be generated with these two methods. For each of the geometrical methods, a number of exemplar lattices were fabricated that represent the range. For each foam that was fabricated, the shear and normal forces were found for six orientations of the lattice (fig. S1). From this, the average and range of the normal and shear forces were found to indicate the possible anisotropy. For TR, we see that although the range of stiffness that can be spanned is lower than that of SP, the change in properties is approximately linear when blending from one lattice cell to another (Fig. 3 and fig. S2). For SP, two methods are shown in Fig. 5, directional superposition programming (DSP) and translational superposition programming (TSP). DSP, shown in fig. S6, offers the largest range of base stiffness, and as the degree of superposition increases, the stiffness increases; however, this does decrease the extent to which the unit cells can show anisotropic behaviors. For TSP, we see that, by increasing the extent to which the unit cells are connected, we can dramatically increase the shear modulus and change the directionality of the anisotropy (fig. S7). This demonstrates how unit cells can be designed with a single base material to span the range of stiffnesses seen tissues seen in animals, from soft through to more structural ones. In total, with these methods we can generate over a million unique cell types, spanning from 25 to 300 kPa.

In the remainder of this section, we first introduce these two geometrical programming methods, the range of properties that can be achieved, and the musculoskeletal structures that can be fabricated through these. Specifically, we look to imitate the range of musculoskeletal structures seen in an elephant, including the soft muscular trunk, and the load-bearing legs and feet (Fig. 1, A and B).

### Topology regulation

For the TR method, we continuously blend between two base unit cells: bcc and XCube. These have been chosen as they have substantially different stiffness properties. The bcc structure has cubic symmetry, which results in quasi-isotropy, whereas the XCube lattice cell is highly anisotropic in both the compressive and shear stiffness, a consequence of its directional structures, as shown in Fig. 3B. Despite these differences, the two structures share topological similarities, which means it is possible to transition smoothly between them through adjustments in geometric parameters. We introduce a parameter, the topology index (*TI*), which describes the ratio of the blending between the two cell types. As illustrated in Fig. 3C for our case of the bcc and XCube, this index directly regulates the inner length at which the switch between the two cells occurs. A *TI* of “1” corresponds to fully XCube, whereas a *TI* of “0” results in a bcc cell. This blending can occur over any number of unit cells.

As we adjust the TI, the properties of the unit cell and its anisotropy change dramatically. The bcc structure exhibits uniform compressive stiffness across all three directions. However, as the structure transitions to XCube, compressive stiffness increases in all directions, with a notably greater increase in the *z* direction compared to the *x* and *y* directions. Simultaneously, the shear stiffness also transitions from being uniform is the different orthogonal axes to anisotropic, with a 40% increase in the *z* direction and a reduction of 85% in the other directions. This geometrical method expands the range of achievable unit cell properties by combining two base cells. It also allows for spatial blending between the two unit cells, enabling the creation of structures with embedded bending profiles.

#### Muscular structures from the TR method

To demonstrate how the TR method can be used to program the deformation of muscular structures, we have developed three tendon-driven structures, which leverage TR to achieve bending, twisting, and helical rotation (Fig. 4 and fig. S11). The design of these structures is predicated upon the optimization framework (details in the Materials and Methods section, Foam structure optimization) and previously known stiffness profiles required for such bending structures (*42*).

The first bending structure exhibits radially varying stiffness, with a bcc configuration at the outer radius transitioning to a higher stiffness XCube structure at the center, regulating the *TI* from 0 to 1 radially. Tendons in the outer radius of the structure and anchored at the top of the structure can initiate bending. This design leverages the low axial stiffness and high deformation ratio of the outer bcc cell (Fig. 4A, ii), whereas the higher relative axial stiffness at the center enhances bending deformation (*42*). This gradient-based structure can achieve a maximum bending angle of 69.6°, which is 30% greater than the same structure that is developed with a nongradient bcc lattice module (Fig. 4A, iii and iv, and fig. S4A).

The twisting module is designed with a reverse radial topology field, where the XCube configuration is at the outer radius that transitions to a bcc structure at the center (Fig. 4B, i). This gradient geometry provides low shear stiffness and high compression stiffness in the outer region, which transitions to a high shear stiffness and low compression stiffness at the center. A twisting pre-offset is applied to the structure to convert compressive force to shear stress within the lattice cell. A tendon is routed through the axial center of the structure. When actuated, the applied compression force generates a twisting deformation with shearing deformation at the outer regions transitioning to compressive deformation at the center (Fig. 4B, ii). A twisting module with varied height (Δ*H*) can achieve a twisting angle of 78.1° (Fig. 4B, iii and iv), whereas the nongradient bcc lattice module with the same twisting pre-offset only generates a reverse twisting angle of −2.6° (fig. S4B).

By regulating the topology field both radially and axially, a helical topology field can be formed, as shown in Fig. 4C(i). In this configuration, the bcc structure with lower axial stiffness at one side transitions to an XCube structure with higher axial stiffness at the other side. This transition direction varies along the axial length, rotating counterclockwise. When the central tendon is pulled, the compression force generates a helical rotation deformation based on bending deformation, with the direction varying along the axis (Fig. 4C, ii and iii, and fig. S4C).

### Superposition programming

Unlike muscular organs such as the elephant trunk, skeletal systems exhibit a broader range of stiffness and anisotropy, along with more discrete modalities and movements. To replicate these behaviors, our two SP methodologies, DSP and TSP, are used. These methods enable regulation of geometric anisotropy and connectivity across various bcc and XCube variants. These geometric methods can be used independently or synergistically, enabling an extensive range of stiffness and anisotropy of lattice cells.

#### SP method of the lattice cell

Superposition is based on the concept that for unit lattice cells, such as the bcc or XCube; by varying their orientation or translation, their directional stiffness is varied. A number of different unit cells can then be superimposed to create new unit cells; we term this SP method.

First, we examine the role of changing a cell’s orientation. The bcc lattice cell, with its orthogonal uniform stiffness, lacks mechanical directionality. Therefore, its orientation is not varied and is designated with a directionality of “0,” indicating the absence of mechanical directionality. In contrast, the XCube cell exhibits pronounced mechanical anisotropy, allowing for three distinct mechanical properties we define at “1,” “2,” and “3,” when there are respectively aligned with the *x*, *y*, and *z* axis. In the “1” configuration, the cell has the highest axial stiffness in the *z* axis (Fig. 5A, i). Further variation in the stiffness properties of a unit cell can be achieved by translating of the lattice cell. By translating the lattice cell by half a cell length in different directions, eight variants can be generated for each XCube lattice cell (Fig. 5A, ii). Each of these has a distinctly different directionality.

Building on using orientation and translation to change the properties of a unit cell, we can superimpose these cells to from a wider range of combinations. With these combinations of base unit cells with varying rotation and translation, an extensive range of new lattice cells with a wide range of mechanical properties can be produced (Fig. 5A, iii). Using this method, a lattice cube with up to four superimposed unit cells (a quadruple cell) can yield around 4 million possible configurations. This number increases over 75 million for the quintuple cell (table S1).

By superposing the lattice cell with different directionality, the stiffness and anisotropy can be varied. As shown in Fig. 5B, comparing the original XCube lattice cell with the directionality of “1,” by superposing lattice cells with the directionality of “2” and “3” (DSP-ii), the Young’s moduli in all three axes are enhanced, whereas the *z* axis has the lowest elastic modulus instead of the highest one as the original XCube cell (DSP-i). In contrast, the shear modulus *G_xy_* becomes the lowest from the highest one of the initial XCube cell, whereas the shear modulus *G_yz_* and *G_xz_* are increased more than 10 times. When XCube lattice cells are aligned in all axes (DSP-iii), the Young’s and shear moduli are uniformly enhanced across all three axes. Further combining these with a bcc lattice cell of directionality “0” (DSP-iv) results in an additional increase in both Young’s and shear moduli while maintaining their uniformity across all directions.

By introducing unit cells with various translations to each cell type, the connectivity of lattice cells can be substantially regulated, enabling the modulation of more variable anisotropy and stiffness (Fig. 5C). For example, using the same directional combination of “0,” “2,” and “3,” and altering the translations of each lattice cell type results in lattice cells exhibiting progressively increased connectivity (Fig. 5C, iv). This adjustment enhances both the Young’s modulus and the shear modulus. Specifically, the Young’s modulus increases up to threefold along the *y* axis, and the shear modulus enhanced to eightfold in the x-y direction compared to the weakest configuration. In addition, lattice cell TSP-i demonstrates its highest Young’s modulus and shear modulus in the *x* and *y*-*z* directions while exhibiting the lowest values in the *z* and *x*-*y* directions. In contrast, lattice cell TSP-ii achieves more uniform Young’s moduli across all three directions and similar shear moduli in the *y*-*z* and *x*-*z* directions, attributed to the consistent connectivity of the XCube cell in the “2” and “3” directions. Moreover, in both lattice cells TSP-iii and TSP-iv, the increased connectivity of the bcc cell enhances the Young’s moduli along the *x* and *y* axes to twice that of the *z* axis. Lattice cell TSP-iii shows more uniform shear moduli across all directions, whereas lattice cell TSP-iv exhibits higher shear moduli in the *y*-*z* and *x*-*z* directions.

#### Skeletal joints from the SP method

**Fig. 6.**
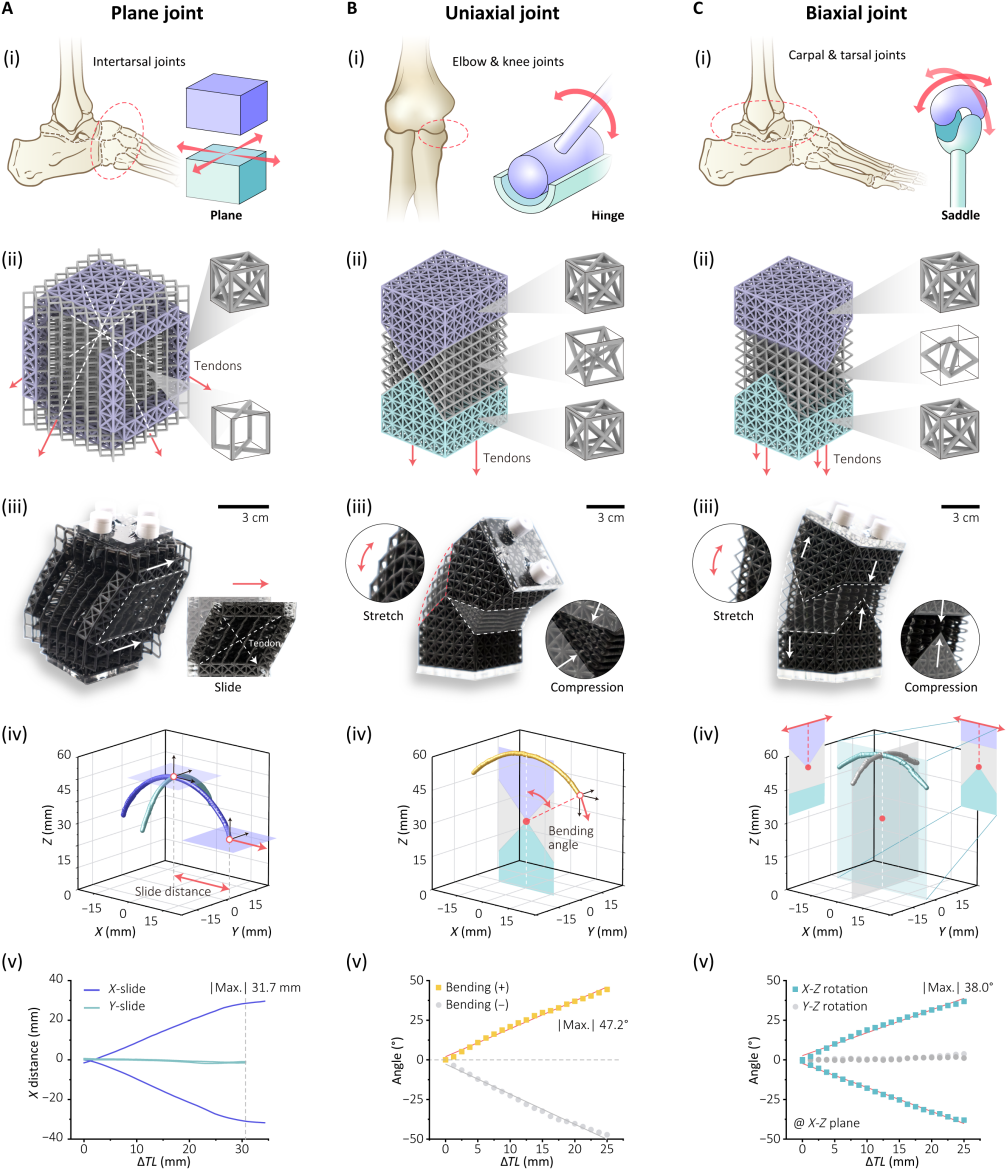
Superpositional-programmed skeletal joints for multiple movements. (**A**) Plane joint for gliding. (**B**) Uniaxial joint for bending. (**C**) Biaxial joint for bidirectional bending. (i) Schematics and mechanisms of joints in biological systems. (ii) Schematics of skeletal joints and tendon layout. (iii) Experimental deformation of movements. (iv) Workspace of skeletal joints. (v) Deformation degrees of skeletal joints with varied tendon length.

To demonstrate the use of the DSP method to mimic skeletal movements, three tendon-driven skeletal joints have been designed to achieve gliding, uniaxial, and biaxial motions, as shown in Fig. 6 and fig. S9. These joints capitalize on the substantial variations in stiffness and anisotropy afforded by SP and the optimization methodology for their discrete distribution (details in the Materials and Methods section, Foam structure optimization).

The first gliding, plane joint is inspired by the intertarsal joint. This structure uses a bidirectional shearing mechanism by embedding rigid, discrete, parallel “bones” of superposed lattice within the soft tissue of the XCube lattice, which exhibits a low shear modulus in the *x*-*z* and *y*-*z* directions (Fig. 6A, ii). Two pairs of tendons are embedded in two orthogonal planes, each pair comprising two antagonistic tendons routed in an “X” shape and actuated by a single motor, as shown in Fig. 6A(iii). When the motor is actuated, one tendon is stretched and its antagonist is released; as a result, the upper surface of the joint glides in the corresponding direction. Reversing the motor’s action causes the joint to glide in the opposite direction. The maximum gliding distances in the *x* and *y* directions are 30 and 25 mm, respectively (Fig. 6A, iv and v, and fig. S10A).

Uniaxial joints are widely seen in musculoskeletal structures, such as elbow or knee joints. The uniaxial joint is constructed using a quadruple superposed lattice cell as the rigid hinge structure and a double superposed lattice cell as the soft ligament (Fig. 6B, ii). The quadruple superposed lattice has substantially higher stiffness in all dimensions, functioning as the bone in the skeletal system. In contrast, the double superposed lattice, characterized by low stiffness along the *z* axis and high stiffness in other dimensions, can undergo high stretching and compression deformation ratios while providing sufficient support and constraint for joint movement (Fig. 6A, iii). Two tendons are embedded on each side of the joint. When one tendon is stretched and the other is released, the joint bends in the corresponding direction. Reversing the actuation causes the joint to bend in the opposite direction (Fig. 6B, iv). The maximum bending angle in both directions can reach up to 50° (Fig. 6B, v, and fig. S10B).

The final joint we mimic is a biaxial joint such as the carpal or tarsal joint, which offers 2D rotation over a saddle joint. The biaxial joint uses the same quadruple superposed lattice cells to construct the rigid “bone” structure; however, these “bones” are oriented in orthogonal directions and interconnected through a bcc lattice. The bcc lattice, characterized by low axial stiffness and high shear stiffness, provides the necessary flexibility and constraint to facilitate universal bending while effectively preventing undesired twisting deformation. Four tendons are evenly distributed and routed in four directions (Fig. 6C, ii). By selectively tensioning or releasing these tendons according to a specific actuation principle, the joint is capable of bending in any direction (Fig. 6C, iii and iv). This universal joint achieves a bending angle of ~40° in all directions, as illustrated in Fig. 6C(v) and fig. S10C.

### Soft/rigid musculoskeletal elephant robot

The elephant has both a soft, dexterous trunk and rigid, strong legs, enabling it to perform delicate manipulations while supporting its heavy body (Fig. 1A). The trunk, primarily composed of muscles and devoid of bones, consists of over 40,000 muscles, which results in its highly dexterous behaviors. In contrast, its straight, pillar-like legs with large dense bones provide strong support and stability. The range of muscular and skeletal structures in an elephant provides inspiration for creating a robotic system, which requires a range of properties and structural types. To create an elephant-inspired robot with a soft, dexterous robotic trunk and four load-bearing legs, we use both the continuous TR method and discrete SP method based on two basic bcc and XCube lattice cells.

#### Elephant trunk

**Fig. 7.**
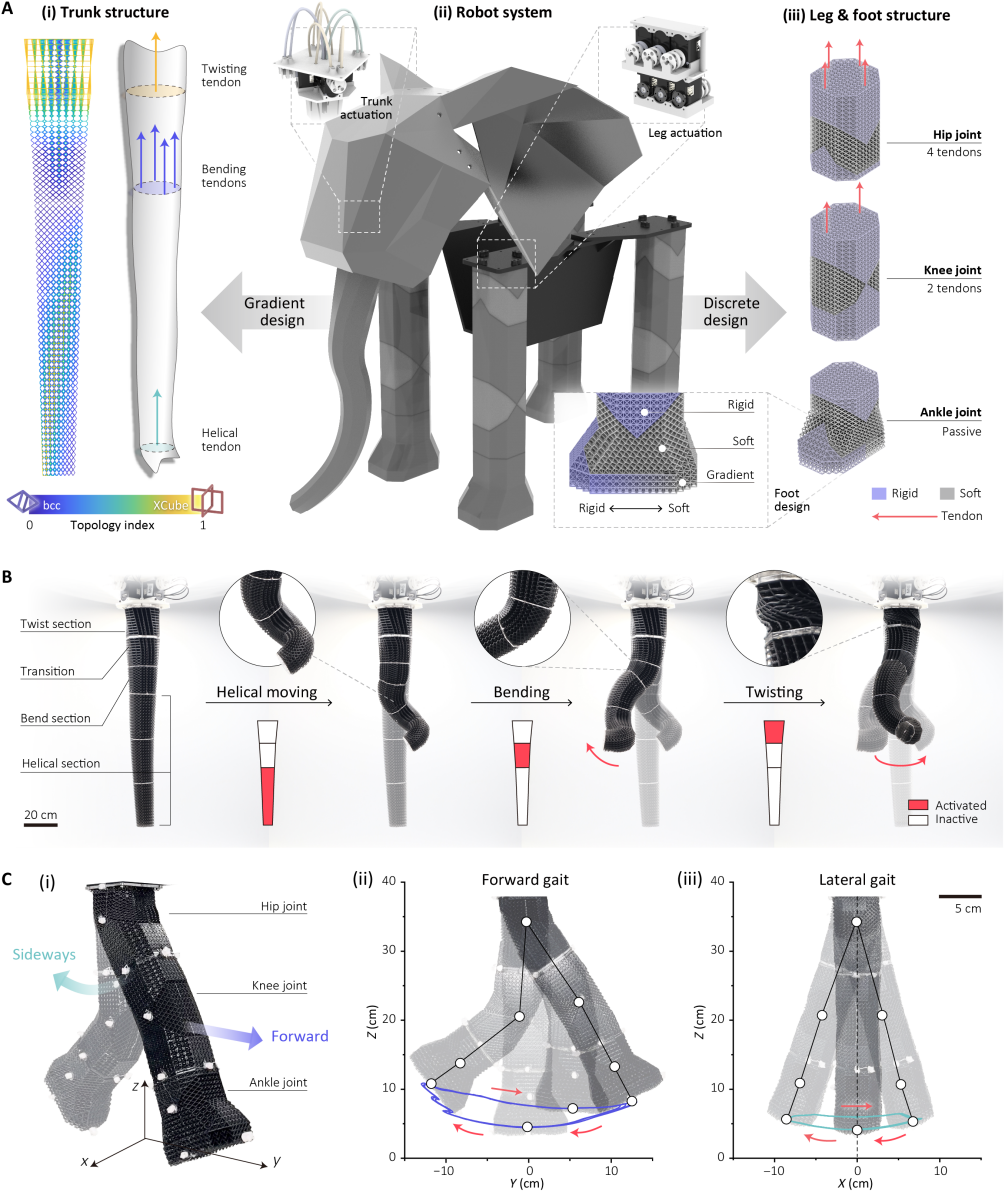
Design of the elephant robot. (**A**) Structure design of the elephant robot. (i) Gradient lattice structure of the trunk. (ii) Robot system with motor actuations. (iii) Discrete lattice structure of the leg and foot. (**B**) Combined moving performance of the elephant trunk. (**C**) Schematic showcasing the two typical gaits of the elephant leg, including forward gait (ii) and lateral gait (iii).

The elephant trunk has the capacity to bend, twist, and wind in a helical structure. To imitate this range of deformation, the trunk is fabricated from lattices designed using the TR method, allowing continuous and versatile deformation without the need for numerous actuation tendons, as shown in Fig. 7A. It has three key parts: twisting, bending, and helical sections, which combine to give a wide range of trunk-inspired motions (movie S1).

The three sections leverage similar *TI* fields proposed for the continuous deformation modules in Fig. 4; however, the topology fields have been modified to ensure smooth transitions between each section, as illustrated in Fig. 7A. The shape of the entire trunk has been tapered, and the lattice cell size and beam thickness of each section have been adjusted to enhance load capability and dexterity (Fig. 7, A and B).

Located at the base of the trunk, the twisting section has the largest lattice cell size and beam thickness to enable it to bear the self-weight of the entire trunk. One single, centrally routed tendon can introduce twisting into the trunk. In the bending section, four tendons are evenly distributed around the cross plane and driven by two motors to provide bending in two axes. The helical section is placed at the tip of the trunk, which has the smallest lattice cell size and beam thickness to allow for more delicate operations under lower tendon forces.

The structural adjustments and variation in stiffness throughout the structures enable a degree of functional independence across the sections, despite the coupling of actuation forces due to the tendon routing. As shown in Fig. 7B, actuation of the helical and bending sections has minimal influence on the deformation of the twisting section due to the much lower stiffness of these sections. The twisting section remains unaffected until its own tendon is activated. This decoupling effect enhances the trunk’s overall functionality and control (figs. S13 and S14).

#### Elephant legs

The straight, pillar-like legs of elephants provide effective support and stability for their heavy bodies. Inspired by this design, an elephant leg structure was developed in our study, featuring a straight configuration constructed using the discrete lattice joints described in the previous section.

The lattice-based elephant leg incorporates two active joints and one passive joint. The hip joint, driven by two motors and four tendons routed in four directions, provides two degrees of freedom (DoFs) for flexion/extension and abduction/adduction movements, as shown in Fig. 7A(ii and iii). The knee joint, actuated by a single motor with two antagonistic tendons, enables flexion/extension movement. The ankle joint serves as a passive joint, adjusting the foot’s orientation in response to environmental interactions.

As the tendons of the knee joint are routed through the hip joint, the hip joint is designed to have a higher bending stiffness. This design enhances stability and minimizes the coupling effects caused by forces transmitted through the hip tendons, ensuring better mechanical decoupling and overall joint performance.

As illustrated in Fig. 7C and movie S2, the robot elephant leg with three DoFs supports both forward and lateral walking gaits. The forward gait consists of four stages: first, the hip and knee joints flex backward to lift the foot; next, the hip joint extends forward, followed by the extension of the knee joint. Subsequently, both the hip and knee joints return to their original straight configuration (Fig. 7C, ii). The lateral gait comprises five stages, as shown in Fig. 7C(iii), to initiate movement to the right; the hip joint first bends to the left. The hip joint then extends, and the knee joint flexes, shortening the leg’s overall length. Following this, the hip joint moves to the right side, and the leg elongates as the hip joint extension and knee joint flexion are released. In the final stage, the hip joint returns to a central lateral position. The robot’s leg gait demonstrated long-term stability and positional accuracy in repeatability tests (fig. S17).

#### Integrated lattice musculoskeletal robot

**Fig. 8.**
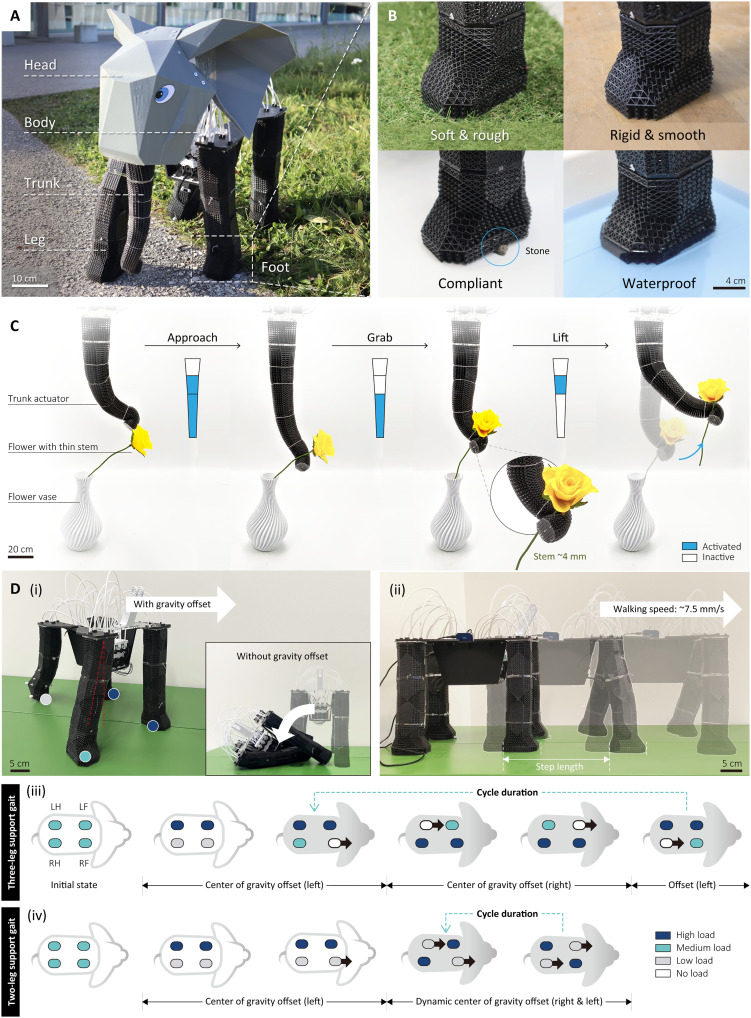
Demonstration of elephant robot’s capabilities. (**A**) Overall appearance and structural composition of the elephant robot. (**B**) adaptability of the elephant foot to various terrains and environments. (**C**) By combining different sections, the trunk can grab a flower from vase. (**D**) Coordinating the center of gravity and gait to achieve biomimetic walking.

The full lattice robot combines the soft robotic trunk capable of dexterous manipulation and the rigid robotic elephant legs capable of static load bearing and dynamic interaction (Fig. 8). With this integrated robot, we can see how passive compliance and actuated behaviors can enable a wide range of adaptive behaviors.

The feet of the elephant robot were capable of passive properties. The feet leverage both TR and SP methods and have been designed to have higher stiffness at the front for weight bearing (fig. S19C) and softer structures around the heel as elephant feet (Fig. 1A). This design enables the feet to be stable and adaptive, as shown in Fig. 8B, where the feet of the robot remain stable across varying terrains and disturbances. In addition, due to the use of the hollow lattice shape and centralized electronics, the robot can also operate in water.

The robotic trunk operates with only four motors while achieving a wide range of motions. Different actuation sections can display specific motion trajectories to approach objects through the sequence in which they are activated and their combination with each other. As shown in Fig. 8C and movie S2, the trunk slid smoothly around the stem of the flower and then clamped the stem without damaging the flower through the contraction of the tip of the trunk. Through the upward helix rotation, the stem can be gently removed from the vase, and then the flower can be stably lifted through the more powerful bending section. By integrating structural compliance with actuation, it adapts to objects of varying sizes and shapes, enabling versatile grasping capabilities (fig. S15). For small or thin objects, the trunk uses the small bending radius of its helical deformation at the tip to clamp objects effectively. For larger objects, the helical section can use its full length or combine with the bending section to wrap around the object’s surface. Through the combination of bending, twisting, and helical motions, the trunk achieves diverse grasping modes, accommodating items such as thin films, slender bars, spheres, and cylinders. The gripping range extends from as small as 0.1 mm to as large as 100 mm, demonstrating its adaptability to a wide range of object scales and geometries. It also performed a high output force that can lift a weight of 500 g, which is more than three times its own weight (~150 g), and still maintain stable dynamic performance under such a high load (fig. S16).

Last, the legs of the robot provide stability and locomotion. The structure of the robotic legs enables the system to support an additional load of 4 kg (fig. S16), over 100% its own self-weight (3.89 kg, shown in fig. S20). The robot can thus support its weight on only three legs, such that it can stably lift its legs in static state (fig. S18B). This functionality also enables the robot to interact dynamically with the environment with the fourth leg, performing tasks such as kicking a ball, facilitating participation in activities like bowling, as demonstrated in Fig. 1E and movie S4. During dynamic walking (Fig. 8D, i), the robot can easily fall due to the frequent switching of gravity distribution. The center of gravity offset mechanism allows the robot to always maintain balance during dynamic movement (Fig. 8D, ii). The robot showcased its capability of walking with various gait patterns (two-leg supporting or three-leg supporting) by continuously adjusting the center of gravity offset to meet the changing leg support situations (Fig. 8D, iii and iv). The walking speed of robot was around 7.5 mm/s with the step length of ~150 mm.

## DISCUSSION

Through the combination of the geometrical methods, TR and SP, we demonstrate how we can realize a range of lattice cell units that can span a range of 20 to 280 kPa in Young’s modulus and 1.38 to 40 kPa in shear modulus. Our method of TR works on generalized lattice shapes, enabling the creation of new lattice structures that offer tunability for much wider stiffness and anisotropy properties. With the combination of TR and SP methods, the properties (stiffness, anisotropy, and connectivity) can vary spatially through structures, either continuously or discretely. This enables behavior programming of both soft and rigid structures. A computational framework for TR and SP has been developed, which can be used for designing motion behaviors through structural programming, integrating continuous regulation and discrete distribution. As a proof of example, we demonstrate our methods by designing a soft-rigid musculoskeletal robot, using only a single material to achieve a wide range of underlying mechanical behaviors through the structural programming.

With two basic lattice cells printed by a single material, we demonstrated the range of stiffness and anisotropy that can be achieved through two methods of geometrical lattice design. Although this covers a large range of the stiffness of different materials seen in bioinspired musculoskeletal structures, stiffer structures (e.g., bone) are still not fully possible. This range could be expanded by either changing the underlying material used in fabrication or by further increasing the range of different geometries that can be used. One key mechanism to do this would be by varying the thickness of the foam lattices, which could substantially increase the possible range of stiffness. Besides, soft or more rigid materials can be used to further extend the range (*43*).

The use of a lattice structure deviates from the more homogeneous nature of typical musculoskeletal structures. We have generated more than 1 million types of cubes based on merely bcc and XCube cells. More types of lattice structures can be introduced for advantageous properties (*44*). Similar to honeycomb and other lattice structures, the strength-to-weight ratio can be very high, enabling lightweight, efficient, and low-inertia robots. The open foam structure is also well suited for motion in fluids where it offers lower resistance while also being inert to fluids. Last, the use of an open, lattice foam allows the potential for including other materials, sensors, and tendons within the structure to provide further intelligence to foams.

Developing tools to enable the optimization of a lattice structure for a required kinematic behavior would make this approach more widely usable. Because of the complexity of the geometries and the deformation modes of the structure, this would most likely require a hybrid approach combining finite element analysis (FEA) (*34*) and data-driven methods (*45*, *46*).

## MATERIALS AND METHODS

### Foam geometry regulation

The design of lattice cell geometry requires encoding of geometric variations with understandable parameters, accounting for millions of possible combinations enabled by the SP method and an infinite range of the continuous TR method.

#### TR method

To encode the TR between the two lattice cell types, bcc and XCube, a unified generic lattice cell configuration is introduced. This configuration shares a similar topology with both lattice types by combining the straight or in-plane beams of the XCube cell with the tilted beams of the bcc cell, as shown in Fig. 2B.

Topology index (*TI*) is proposed to quantify the continuous TR method of the lattice cell geometry. The *TI* is defined as the nominal length of the straight beam along the *z* axis of the generic lattice cell relative to the cell size, expressed as

$$\mathrm{TI}=\frac{L_{1}}{L_{\text{cell}}}$$

where *L*_1_ is the length of the straight beam, and *L_cell_* represents the size of the lattice cell. By varying the *TI* from 0 to 1, the generic lattice cell transitions smoothly from a bcc cell (*TI* = 0) to an XCube cell (*TI* = 1). This regulation enables a continuous spectrum of lattice geometries to be generated, as shown in Fig. 2B.

The continuous topological regulation of different lattice structures can be achieved through the body-centered blending (BCB) method. This approach designates the corner point of one lattice phase as the body center of another. The blending process begins by modifying the host lattice cell through the removal of material around its corner within a cubic space, which is subsequently filled with an intact guest lattice cell. If the host and guest cells are not inherently connected, an initial offset is introduced to align either the host or guest cell, ensuring the formation of a well-integrated lattice structure, as illustrated in Fig. 3.

Both host and guest lattice cells may exhibit anisotropic properties, which can be derived by selectively extracting portions of or incorporating additional elements into common isotropic lattice cells. As the dimensions of the trimming cubic space vary, the phase proportion is correspondingly adjusted, enabling a smooth transition between different lattice structures. This method is applicable not only to anisotropic lattice cells but also to isotropic ones, thereby facilitating seamless connectivity among various commonly used lattice configurations.

#### SP method

To encode the programming, the SP indices for directional superposition programming (DSP) and translational superposition programming (TSP) are introduced.

As previously mentioned, the directional index (*DI*) is used to describe the directionality of a mono cell. Specifically, *DI* = 0 represents the bcc cell, whereas *DI* = 1, 2, and 3 represent the straight beams of the XCube cell oriented along the *z*, *y*, and *x* axes, respectively, as illustrated in Fig. 5A(i). The translational index (*TSI*) is defined as the nominal translation vector of the lattice cell. For each mono cell, the vector is characterized by three parameters, representing the normalized translation displacements along the *x*, *y*, and *z* axes relative to the half-cell length, as shown in Fig. 5A(ii). Using the *DI* and *TSI*, 28 mono cells with distinct directionality and translation configurations can be encoded for each base cell type (i.e., XCube or bcc).

These indices enable DSP and TSP to be applied either individually or in combination to multiple diverse mono bcc or XCube cells, facilitating the generation of a vast number of lattice types (Fig. 5A, iii). Furthermore, by involving five selected topology-regulated lattice cell variants, the DSP and TSP together can generate over 4 million of distinct quadruple lattice cell types.

### Foam structure design

The foam structure is generated using the OpenSCAD code, based on lattice beam position and shape data that are automatically produced in MATLAB following specific design rules.

#### Foam structure definition

To design lattice foam structures ranging from single cells to functionally varied structures, the *TI* field and SP domains must first be defined. Boolean and mapping operations are then applied to different regions to create more complex structures, such as irregular, tapered, or twisted geometries.

The topology field can be represented using either parameter-defined analytical formulas or numerical functions interpolated from discrete sample points. The topology of each cell is determined by the calculated or interpolated value at the body center of the cell.

As SP must be applied to discrete regions at specific locations, the applied domain is first defined using constrained conditions. For example, a domain can be specified by the inequalities |*x*| < *x*_1_, |*y*| < *y*_1_, and |*z*| *< z*_1_. Lattice cells within these domains are then assigned to corresponding SP indices to satisfy design constraints..

#### Foam structure file generation

On the basis of the aforementioned rules and design requirements, MATLAB or other software can be used to generate the OpenSCAD file for the foam structure. The STL file is subsequently produced using the open-source software Hob3l.

### Foam structure optimization

For TR-programmed lattice structures, a continuous *TI* index field is adopted to optimize structural design for different deformation patterns, such as bending and rotation. An axisymmetric distribution with a polynomial form in the radial direction is introduced as the topology index field. A multiobjective optimization approach is used to obtain to achieve a balance between different performance requirements.

For SP-programmed lattice structures, the spatial distribution of different lattice type is optimized with genetic algorithm based on the discrete parameters indicating the lattice type in different regions. For instance, in the design of a bending joint, “rigid” lattice cell numbers of each column were involved as optimize parameters. The bending stiffness was set as the optimize target, and the bending range and axial stiffness are constrained to above a certain level.

All the calculation is realized in MATLAB with the optimization toolbox. The Young’s moduli and shear moduli are obtained by interpolating from the experimental data and based on the local *TI* value.

### Foam fabrication

The lattice foam is 3D printed by the Halot-Mage Pro printer (Creality 3D Technology Co. Ltd., China) with F80 elastic resin (Godsaid Technology Co. Ltd., China). The printing parameters are set as table S2.

### Foam testing and characterization

All tested lattice foam samples for TR and SP methods were fabricated as 20 mm–by–20 mm–by–20 mm cubes with a beam thickness of 0.75 mm, except for the cube samples used for testing TSP programming. For these TSP samples, the beam thickness was reduced to 0.5 mm to prevent beam merging due to the increased thickness.

For compression testing, the lattice cube is rigidly fixed at its base to a stationary platen while a moving platen applies axial displacement at a controlled rate (0.2 mm/s) using a force testing machine. Force and displacement data are recorded via integrated load cells and extensometers. For shear testing, the cube is clamped between dual parallel plates, where the top plate translates horizontally to induce shear deformation while the bottom plate remains fixed, ensuring no vertical displacement to decouple shear and compressive effects. Both tests use 3D-printed lattice cubes with force-displacement curves analyzed to extract elastic moduli. All data were validated through five repeated trials to reduce errors.

Each cube sample underwent three cycles of uniaxial compression loading along the *x*, *y*, and *z* axes and three cycles of shear loading in the *y*-*z*, *x*-*z*, and *x*-*y* planes, as shown in figs. S1, S2, S6, and S7. During testing, some lattice structures exhibited buckling, producing substantial hysteresis effects. To calculate the moduli, only the forward compression or shear data were used to ensure accuracy.

### Elephant robot design

The elephant robot composes of a robotic lattice trunk and four lattice legs, which is actuated by tendons guided with Bowden tubes and driven by servo motors, as shown in fig. S12.

#### Soft robotic trunk design

To enhance the load-bearing capacity and dexterity, the thickness and cell size of the lattice structure are adjusted along the length of the trunk. The total length of the lattice trunk is 322.5 mm, with a radius of 48 mm at the base, tapering to 24 mm at the tip. Each cross-sectional layer consists of an 8 × 8 cell arrangement. The thickness of the lattice beams varies accordingly, measuring 1.3 mm at the base and tapering to 0.5 mm at the tip to provide structural integrity and flexibility.

#### Elephant leg design

As previously mentioned, the leg features two active joints—the hip and knee joints—and one passive ankle joint. The hip joint is designed with higher stiffness than the other joints by using a double superposed lattice structure, rather than the mono bcc lattice, as a “ligament” to connect the rigid “bones.”

The bottom of the foot is constructed using a lattice structure that combines TR and SP methods to achieve higher stiffness at the front and increased softness around the heel. At the front of the foot, two mono XCube lattice cells with directionalities of “2” and “3” are superposed to form a rigid configuration. Toward the heel, one of the XCube lattice cells is modulated into a bcc lattice cell through the TR method. This transition results in a lattice structure composed of a superposition of an XCube lattice with directionality “3” and a bcc lattice with directionality “0,” substantially reducing stiffness along the *z* axis and providing enhanced compliance.

#### Tendon routing

The robotic elephant trunk and legs are actuated using tendons guided through Bowden cables. For the robotic trunk, twisting and rotational deformations are each actuated by individual motors (Dynamixel XC330-M188), whereas universal bending is achieved using two pairs of antagonistic tendons. In each pair, the two tendons are connected to a single motor (Dynamixel XL330-M288) in reverse configurations with pretension.

For the robotic leg, the hip joint is actuated by two pairs of antagonistic tendons, and the knee joint is actuated by one pair of antagonistic tendons. Each pair of tendons is driven by a single motor (Dynamixel XC330-M288 or XL330-M288). All Dynamixel motors are controlled via MATLAB, using the Dynamixel SDK package, as illustrated in fig. S9.

### Motion data collection

The ground truth measurement of the motion of the continuous deformation modules, rigid joints, and lattice legs is collected using six motion capture cameras (Optic Prime 13). The position and attitude information were collected and exported to MATLAB where the data were postprocessed to remove invalid data points.
