# Supplementary material for: Lattice structure musculoskeletal robots: Harnessing programmable geometric topology and anisotropy
Source: Sci Adv. 2025 Jul 16;11(29):eadu9856. doi: 10.1126/sciadv.adu9856 (PMC12266121; doi:10.1126/sciadv.adu9856)
Supplement: Supplementary file 1 — Supplementary Text Figs. S1 to S21 Tables S1 and S2 Legends for movies S1 to S5 [file sciadv.adu9856_sm.pdf]

Science Advances
AAAS

Supplementary Materials for

Lattice structure musculoskeletal robots: Harnessing programmable geometric topology and anisotropy

Qinghua Guan *et al.*

Corresponding author: Josie Hughes, josie.hughes@epfl.ch; Qinghua Guan, qinghua.guan@epfl.ch

Sci. Adv. **11**, eadu9856 (2025)
DOI: 10.1126/sciadv.adu9856

The PDF file includes:

Supplementary Text
Figs. S1 to S21
Tables S1 and S2
Legends for movies S1 to S5

Other Supplementary Material for this manuscript includes the following:

Movies S1 to S5

Supplementary Text

Lattice samples and structure characterization

The compression and shear cyclic loads were applied as illustrated in fig. S1. The TR samples and their corresponding test results are presented in fig. S2. The optimization of continuous structure is introduced in fig. S3. The deformation results of continuous muscular structures are shown in fig. S4, while the motion repeatability of each muscular structure is detailed in fig. S5. The SP samples and their test results are provided in fig. S6 and fig. S7. The optimization of discrete joint is introduced in fig. S8. Test results for the skeletal joints are shown in fig. S9, with their motion repeatability highlighted in fig. S10. Finally, the test setup for both the muscular structures and skeletal joints is illustrated in fig. S11.

Elephant robot design and demonstrations

The elephant robot comprises a robotic trunk, four legs, corresponding motors, and a central body and head structure that securely connect the trunk and legs, as illustrated in fig. S12. Fig. S13 and S14 demonstrate the deformed robotic trunk under separate and combined actuation movements, respectively. The trunk's ability to grasp various objects is shown in fig. S15. The output force and stability of the trunk is illustrated in fig. S16. Fig. S17 showcases the repeatability of the robotic leg. Fig. S18 and S19 depict the load-bearing capability and control of the robotic legs. The overall dimensions and weight of the elephant robot are provided in fig. S20. The walking sequence photos are shown in fig. S21.

Supplementary Figures

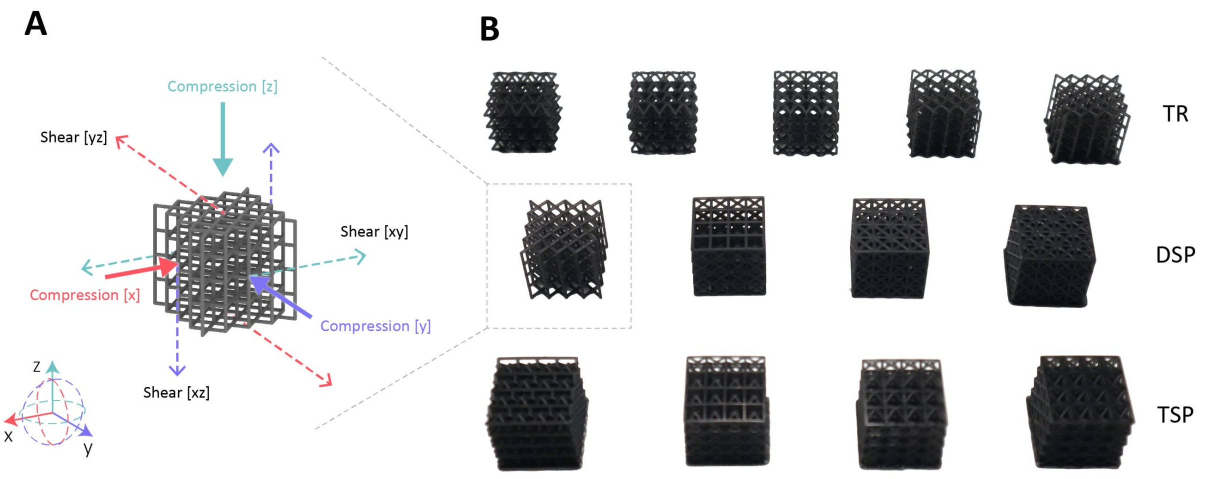

Fig. S1. The diagram of the setup for characterizing the mechanical properties of the lattice cubes. (**A**) Schematic showing the direction of the normal force and shear force applied in the characterization experiment. (**B**) Optical photo of all testing array samples programmed by TR, DSP and TSP methods.

C_x: Compression force in the x direction

C_y: Compression force in the y direction

C_z: Compression force in the z direction

S_{yz}: Shear force in the y-z direction

S_{xz}: Shear force in the x-z direction

S_{xy}: Shear force in the x-y direction

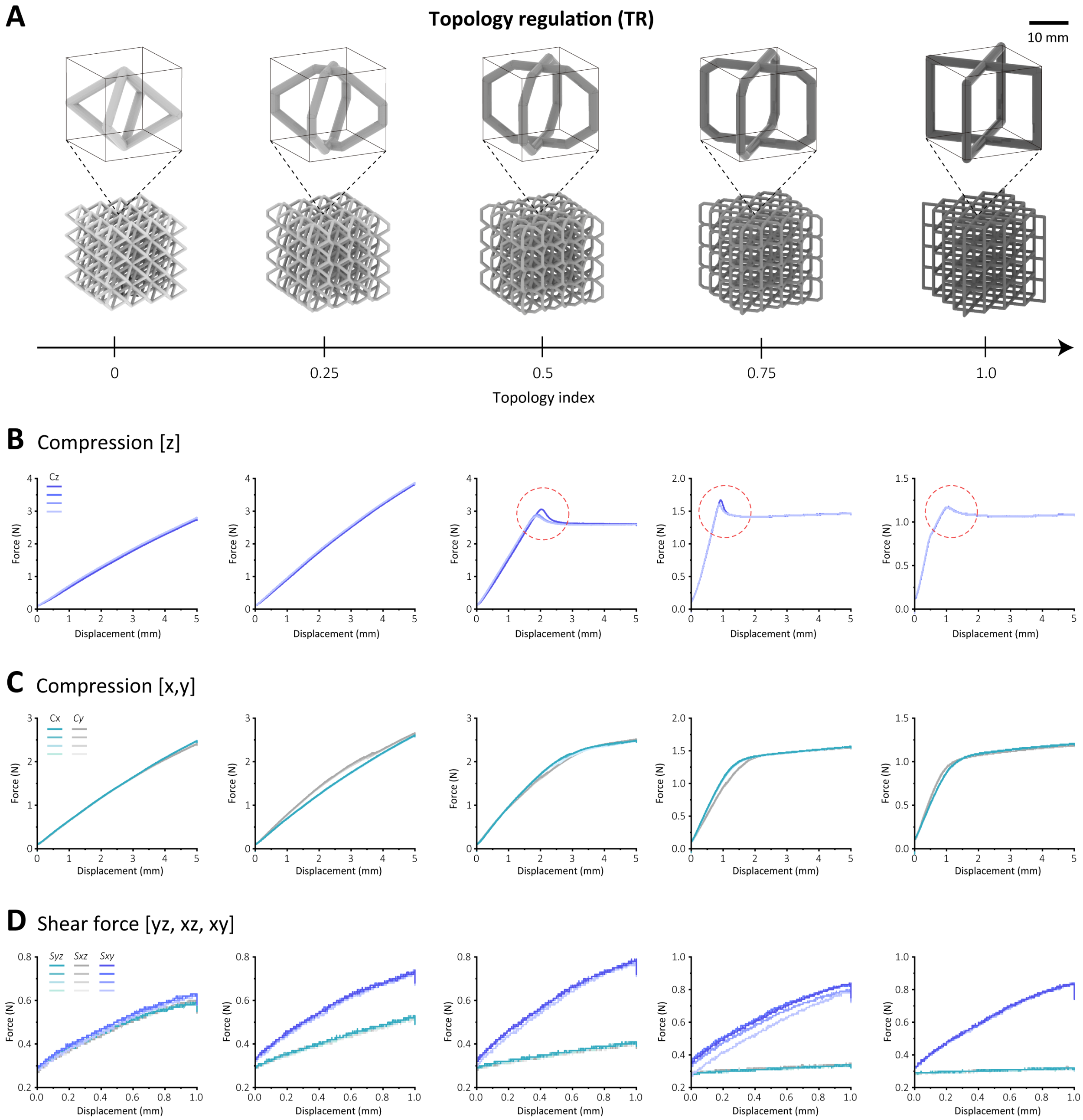

Fig. S2. Characterization of cubes based on TR method. (**A**) Schematic showing topology-regulated cubes, which blend from BCC to XCube. (**B**) Normal force-displacement curve along the z-axis. (**C**) Normal force-displacement curve along the x-axis and y-axis. (**D**) Shear force-displacement curve along y-z plane, x-z plane, and x-y plane.

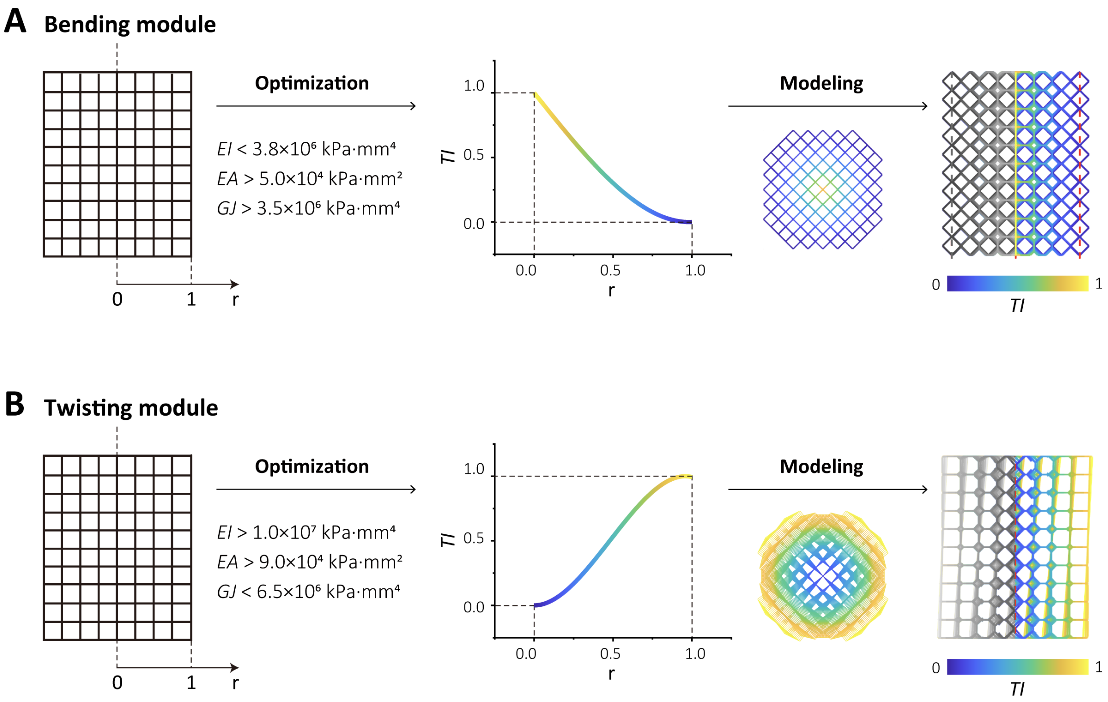

Fig. S3. TR-programmed structure optimization. (**A**) Design of a bending module. (**B**) Design of a twisting module.

In the development of a bending module, the bending stiffness (*EI*) is optimized to be at most 3.8×10^6 kPa·mm^4 to ensure sufficient flexibility. Meanwhile, the axial stiffness (*EA*) and torsional stiffness (*GJ*) are constrained to remain above 5.0×10^4 kPa·mm^2 and 3.5×10^6 kPa·mm^4, respectively, to maintain adequate load-bearing capacity. The resulting *TI* function is given by:

$$TI = 1.00 - 1.56r + 3.40 \times 10^{-6}r^2 + 0.54r^3, \quad \text{for } r \in [0,1]$$

For the twisting module, the torsional stiffness (GJ) is optimized to be at most 6.5×10^5 kPa·mm^4 to ensure sufficient compliance with twisting deformation. Simultaneously, the axial stiffness (EA) and bending stiffness (EI) are constrained to exceed 9.0×10^4 kPa·mm^2 and 1.0×10^7 kPa·mm^4, respectively, to enasure structural stability and sufficient load-bearing capacity. The corresponding *TI* function is:

$$TI = 3.12 \times 10^{-17} + 3.46 \times 10^{-16}r + 3.29r^2 - 2.29r^3, \quad \text{for } r \in [0,1]$$

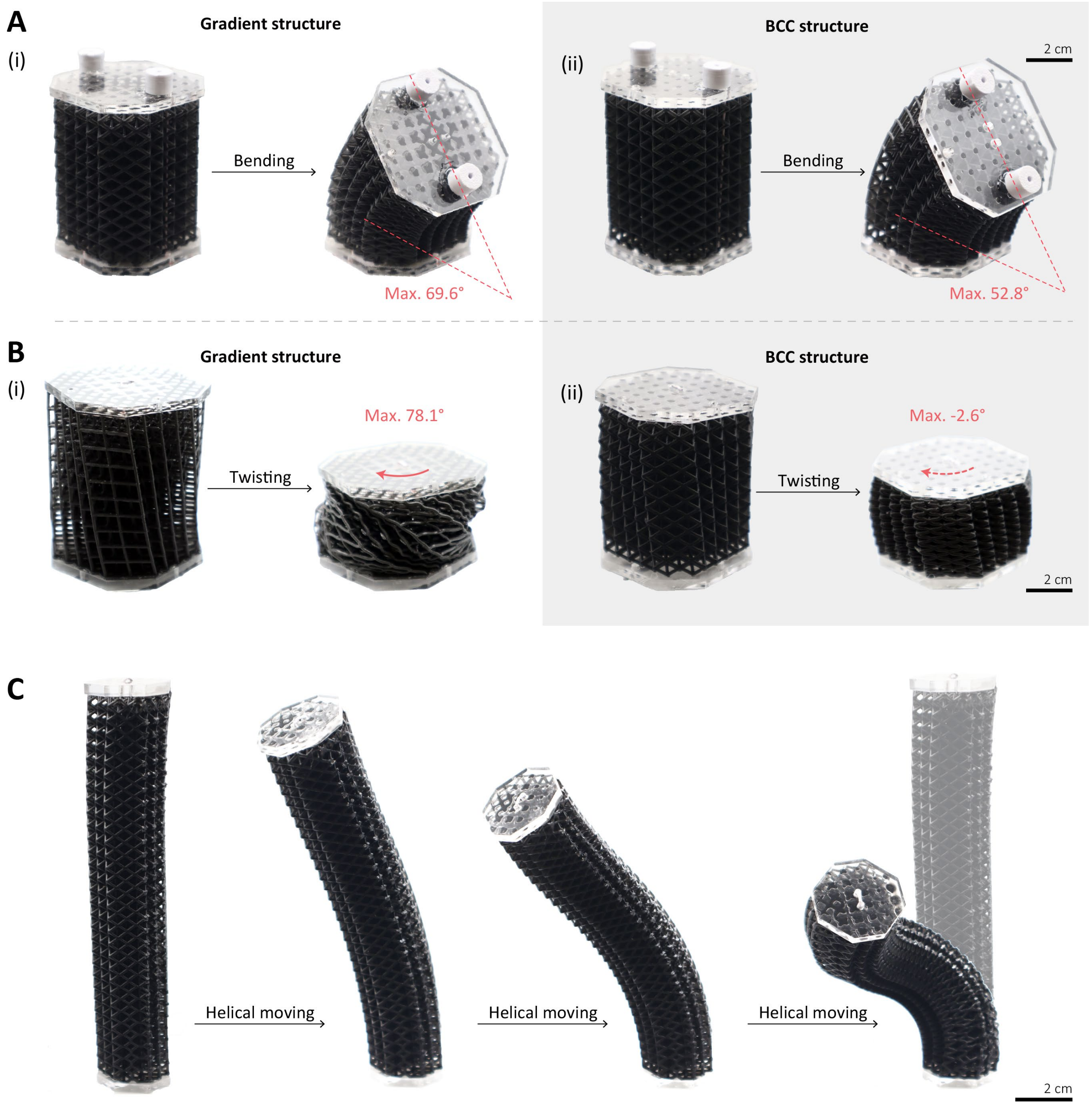

Fig. S4. Movements of muscular structures. (**A**) Bending movement: compare (i) gradient structure and (ii) BCC-based structure. (**B**) Twisting movement: compare (i) gradient structure and (ii) BCC-based structure. (**C**) Helical movement.

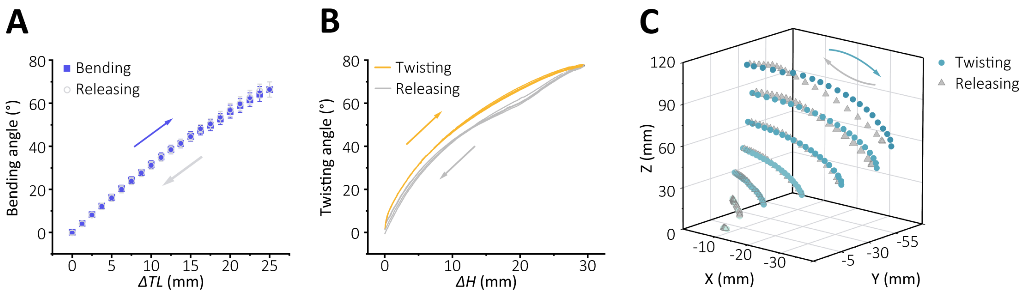

Fig. S5. Moving repeatability of muscular structures. (**A**) Bending movement. (**B**) Twisting movement. (**C**) Helical movement.

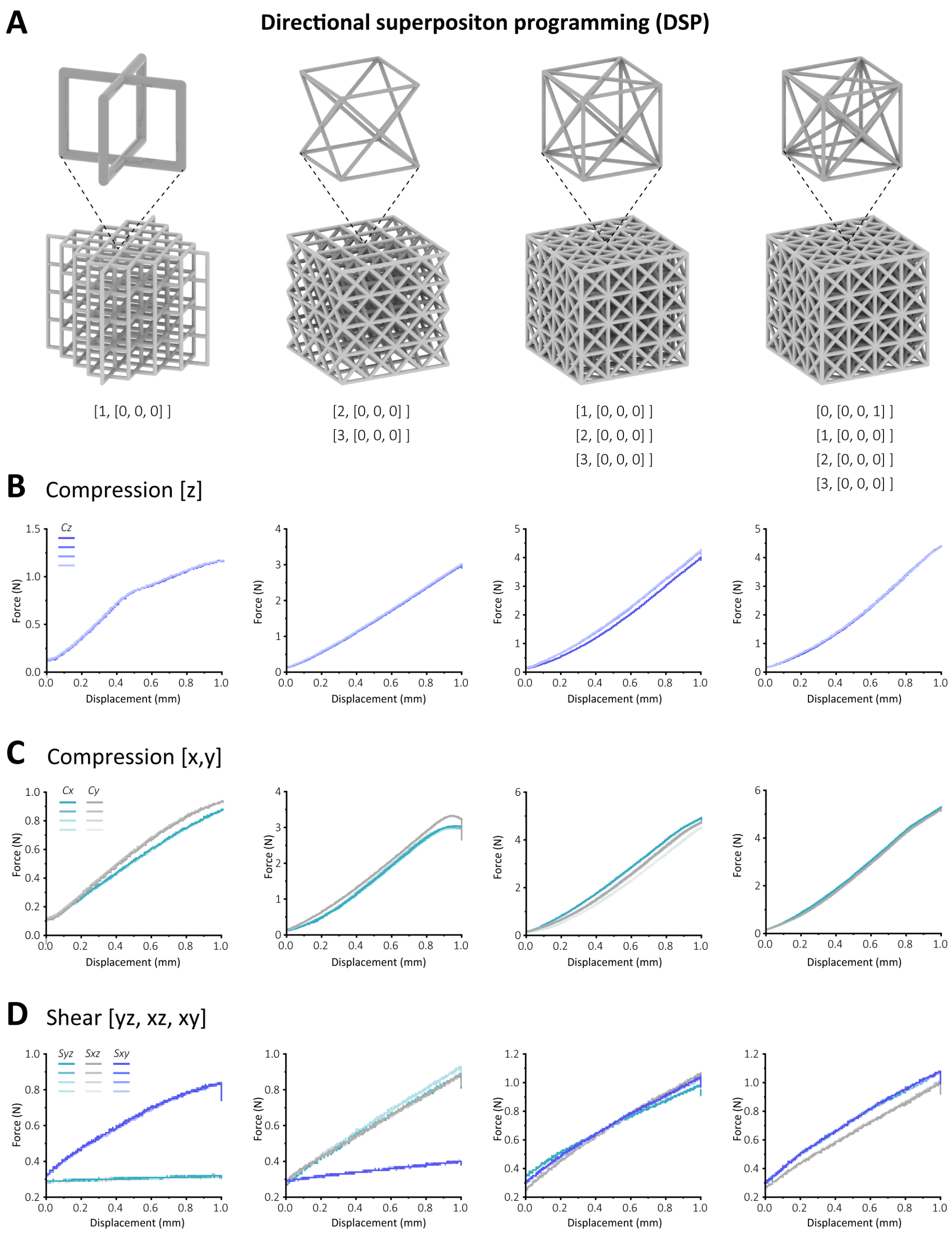

Fig. S6. Characterization of the cubes based on DSP method. (**A**) Schematic showing cubes with increasing degree of superposition. (**B**) Normal force-displacement curve along the z-axis. (**C**) Normal force-displacement curve along the x-axis and y-axis. (**D**) Shear force-displacement curve along y-z plane, x-z plane, and x-y plane.

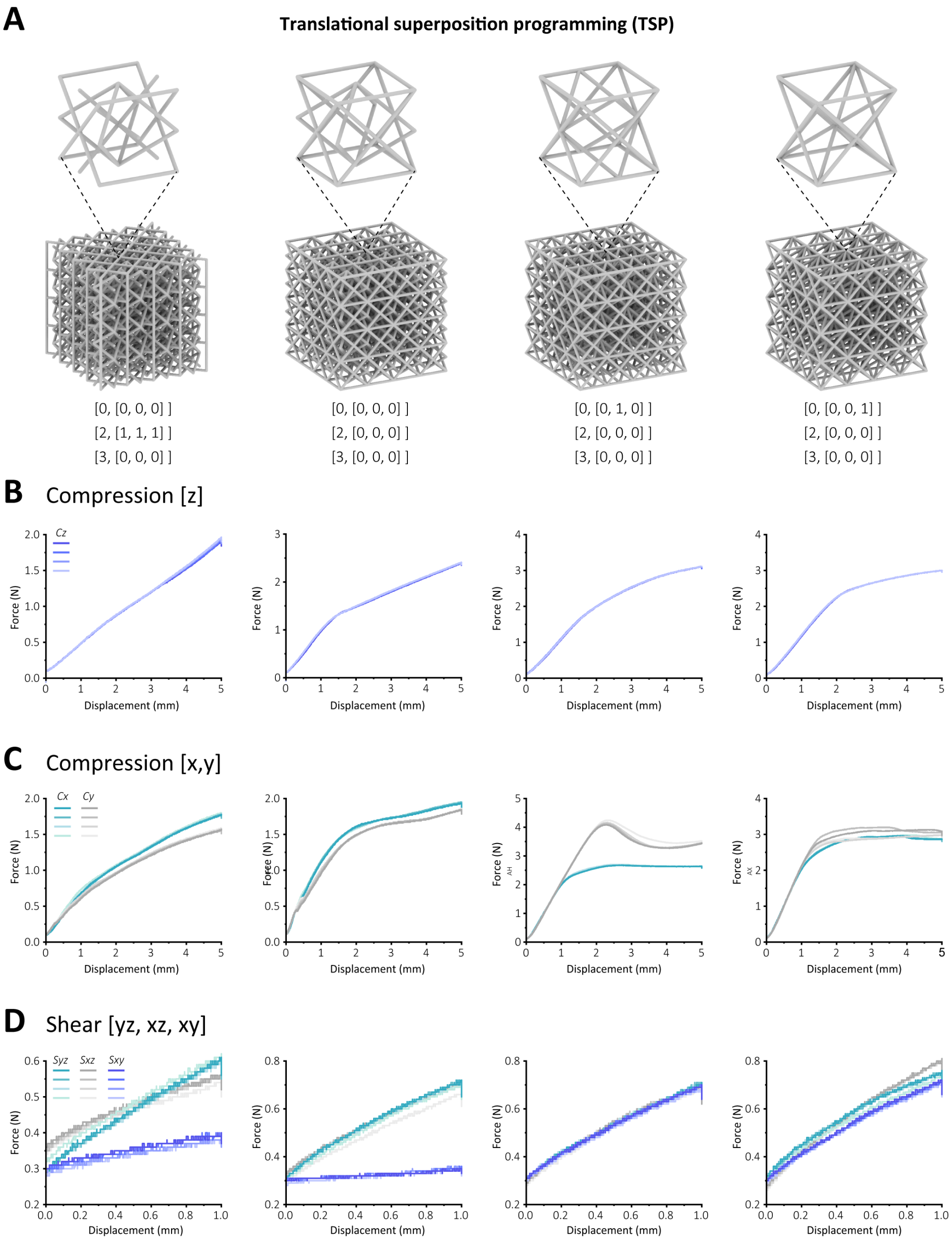

Fig. S7. Characterization of the cubes based on TSP method. (**A**) Schematic showing cubes with increasing degree of connectivity. (**B**) Normal force-displacement curve along the z-axis. (**C**) Normal force-displacement curve along the x-axis and y-axis. (**D**) Shear force-displacement curve along y-z plane, x-z plane, and x-y plane.

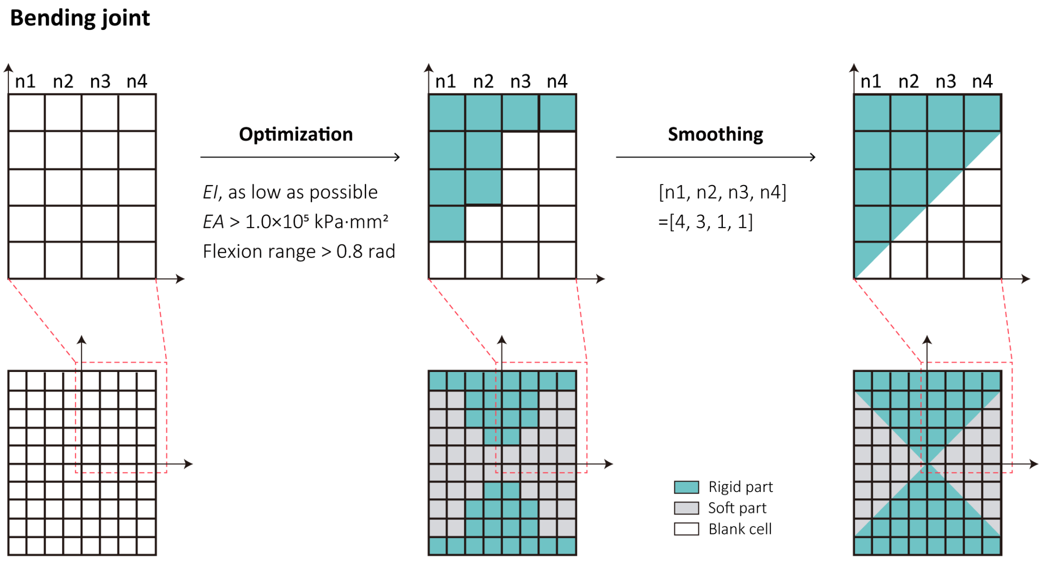

Fig. S8. SP-programmed structure optimization (e.g., design of a bending joint).

For the bending joint design, the bending stiffness (*EI*) is optimized to be minimized as much as possible to enhance compliance with flexural deformation. At the same time, the axial stiffness (*EA*) and flexion range are required to exceed 1.0×10^5 kPa·mm² and 0.8 rad, respectively, to maintain structural stability and sufficient load-bearing capacity. To simplify calculations while considering symmetrical conditions, only one-quarter of the joint is used for optimization. The resulting optimization parameter set is: [4,3,1,1].

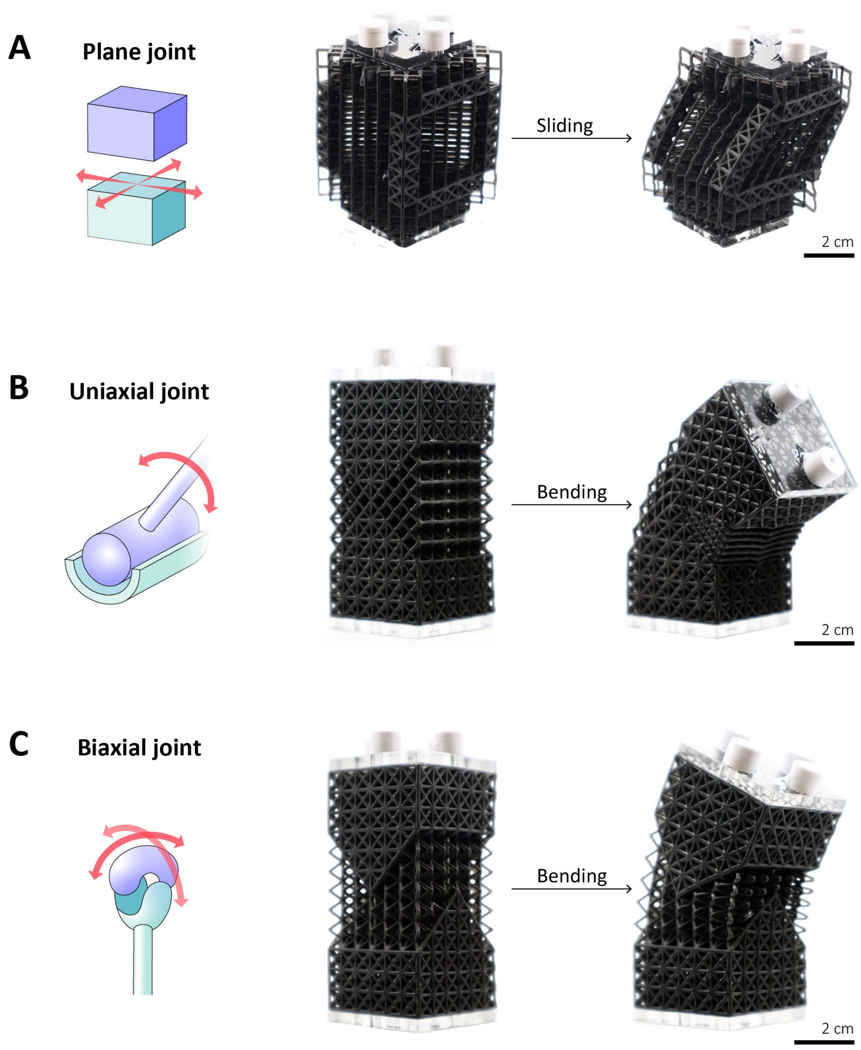

Fig. S9. Bio-inspiration and skeletal joint movements. (**A**) Sliding movement inspired by plane joint. (**B**) Bending movement inspired by uniaxial joint. (**C**) Bidirectional bending movements inspired by biaxial joint.

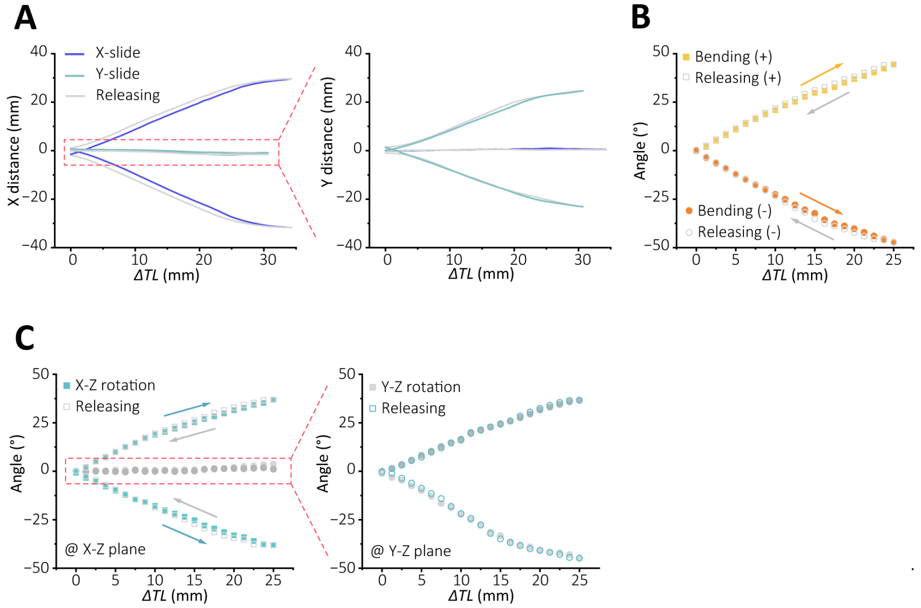

Fig. S10. Moving repeatability of skeletal joints. (**A**) Sliding movement. (**B**) Bending movement. (C) Bidirectional bending movement.

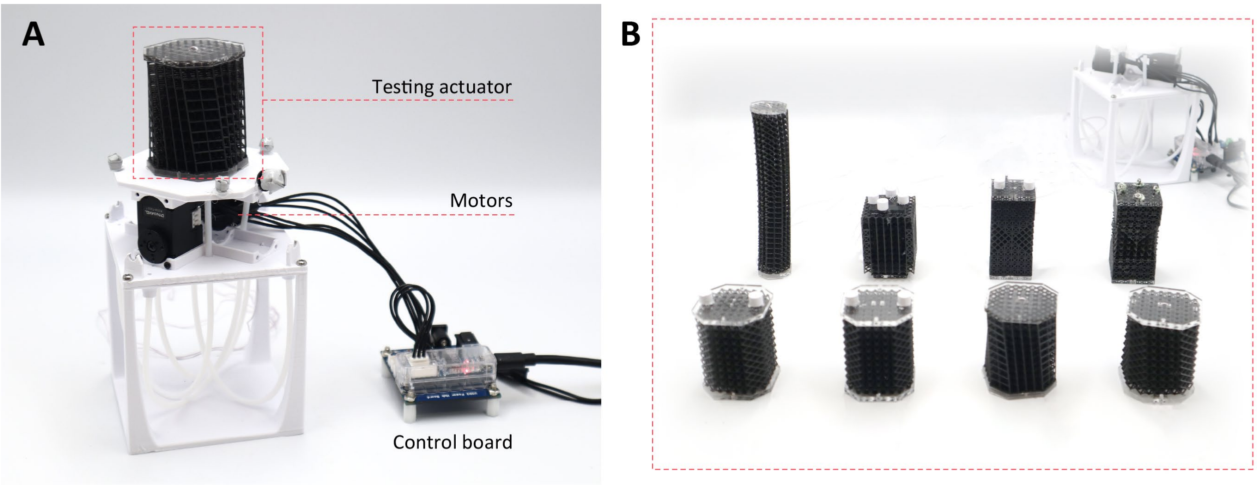

Fig. S11. Set-up for actuating muscular structures and skeletal joints. (**A**) Multiple motors are placed at the bottom of the sample to pull the tendons. The control board is utilized to program and drive different movements. (**B**) Testing samples, including muscular structures and skeletal joints.

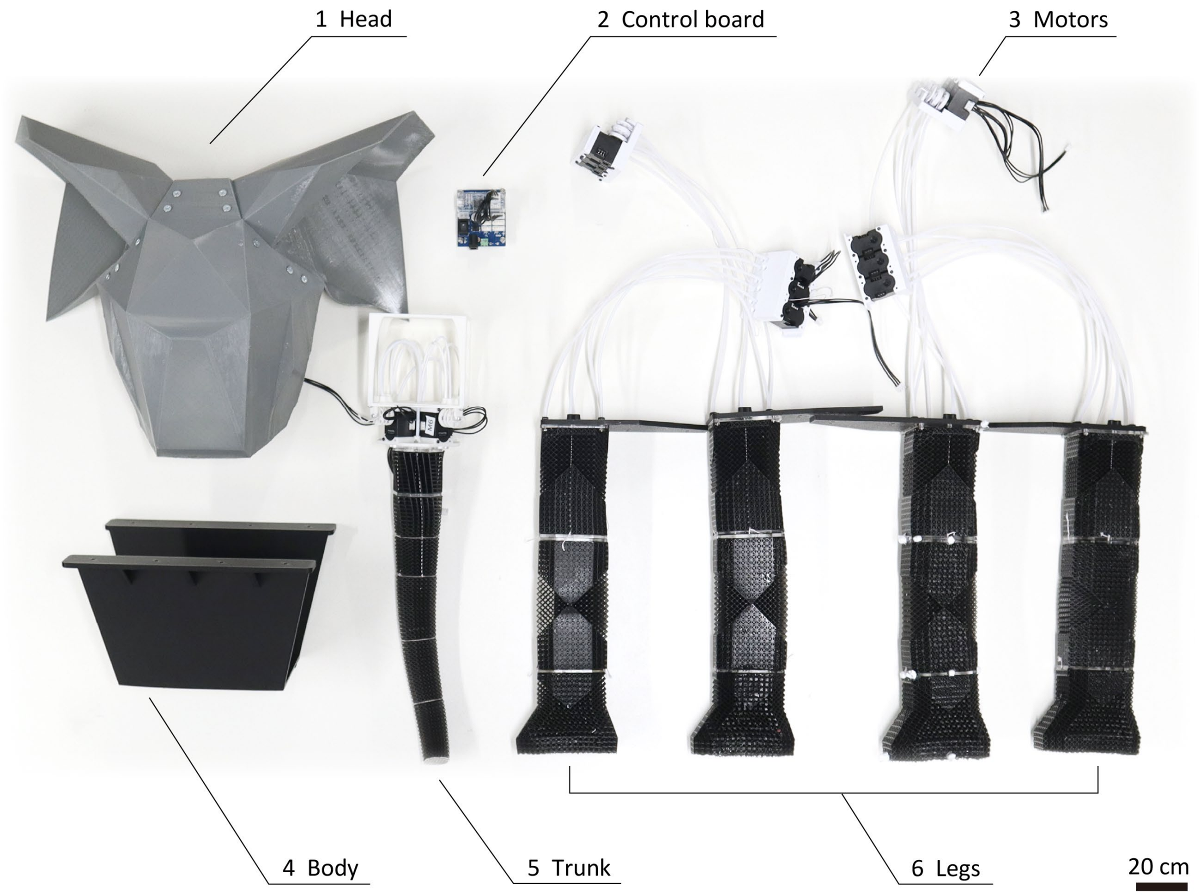

Fig. S12. Components of the elephant robot. 1-TPU elephant head, 2-U2D2 control board, 3-Dynamixel motors, 4-PLA elephant body, 5-elephant trunk, 6-elephant legs.

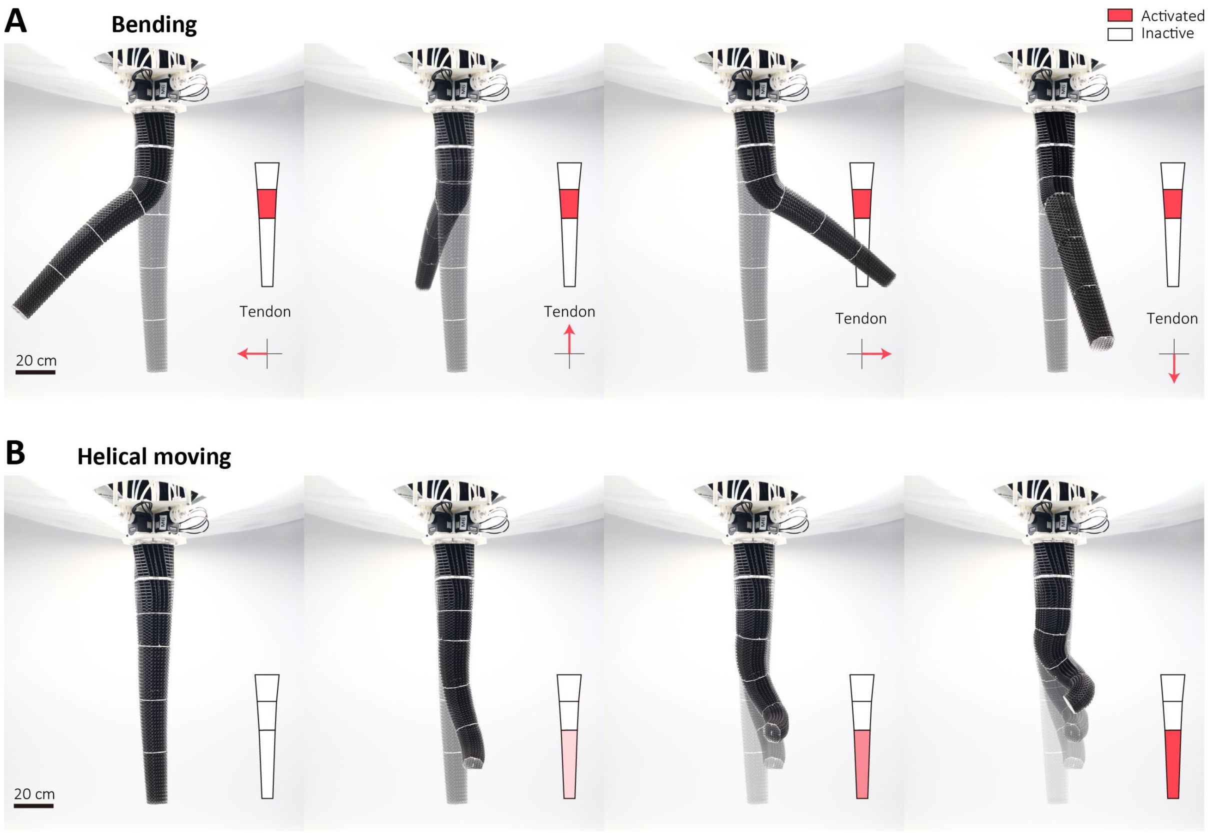

Fig. S13. Elephant trunk actuated by separate movement. (**A**) Bending movements of different directions. (**B**) Helical movements with increasing pulling degree.

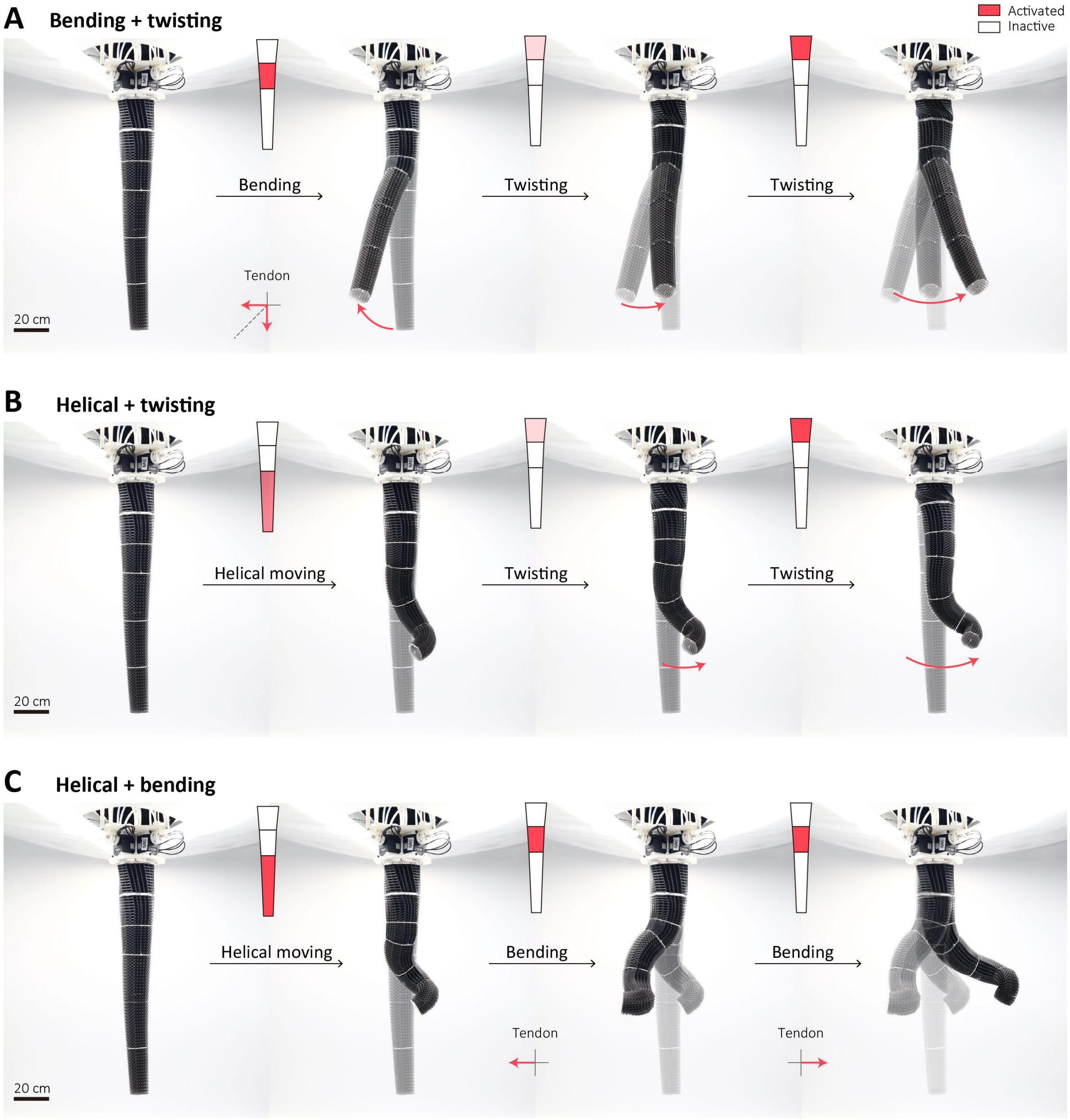

Fig. S14. Elephant trunk actuated by combined movements. (**A**) "Bending + twisting" combination. (**B**) "Helical + twisting" combination. (**C**) "Helical + bending" combination.

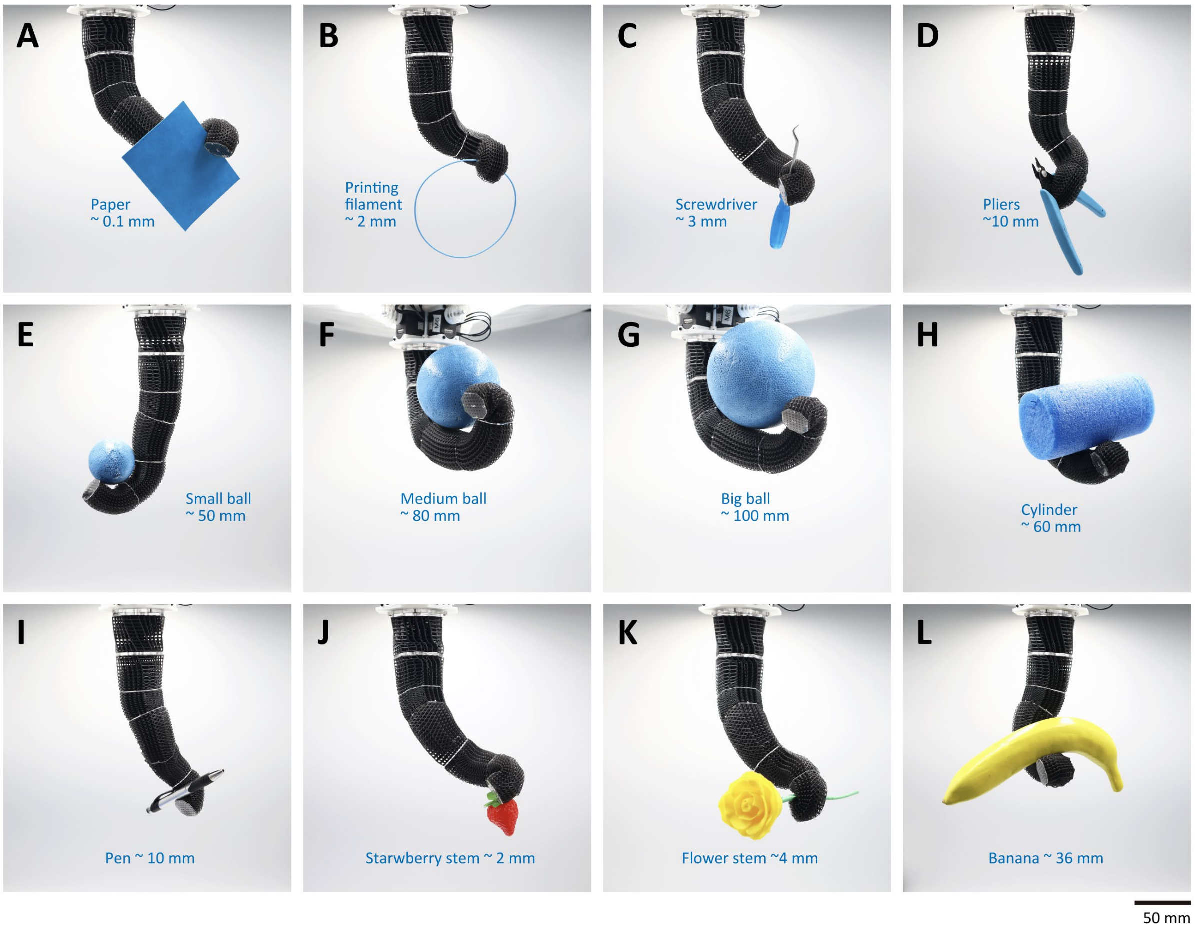

Fig. S15. Elephant trunk grasping objects of a wide range of sizes. (**A**) Paper. (**B**) Printing filament. (**C**) Screwdriver. (**D**) Pilers. (**E**) Small ball. (**F**) Medium ball. (**G**) Big ball. (**H**) Cylinder. (**I**) Pen. (**J**) Strawberry. (**K**) Flower stem. (**L**) Banana.

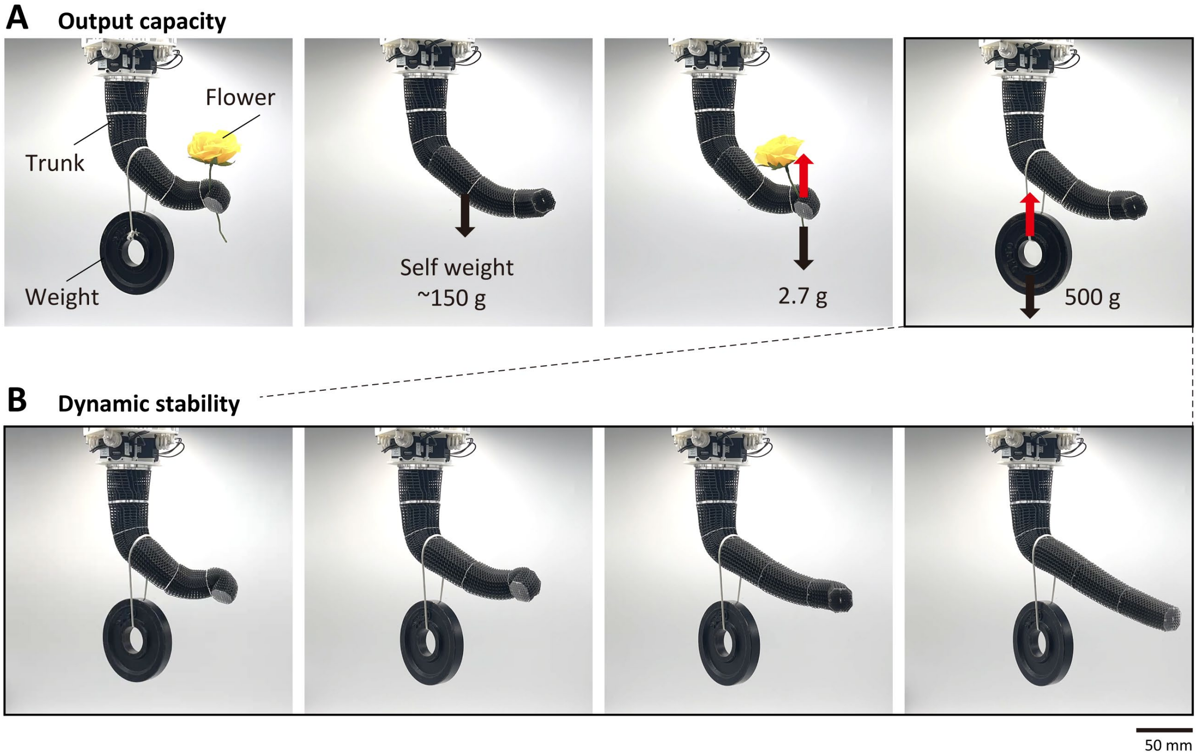

Fig. S16. Output capacity and dynamic stability of the robotic trunk. (**A**) Output capacity. (**B**) Dynamic stability**.**

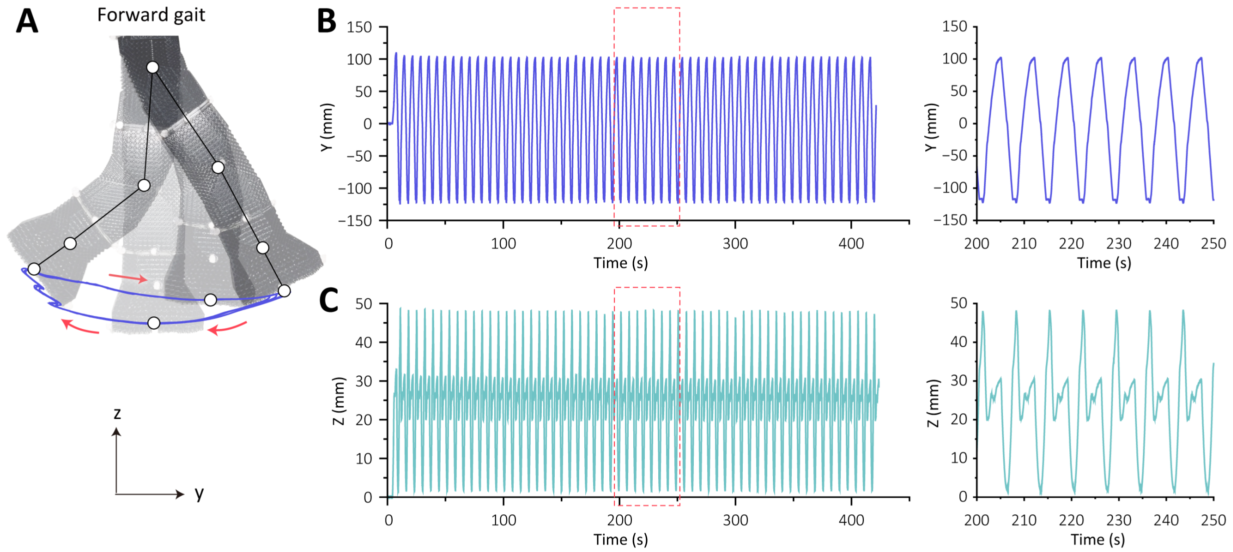

Fig. S17. Repeatability test of the robotic leg. (**A**) Diagram of leg gait movement. (**B**) Moving range at Y axis during gait cycles. (**C**) Moving range at Z axis during gait cycles.

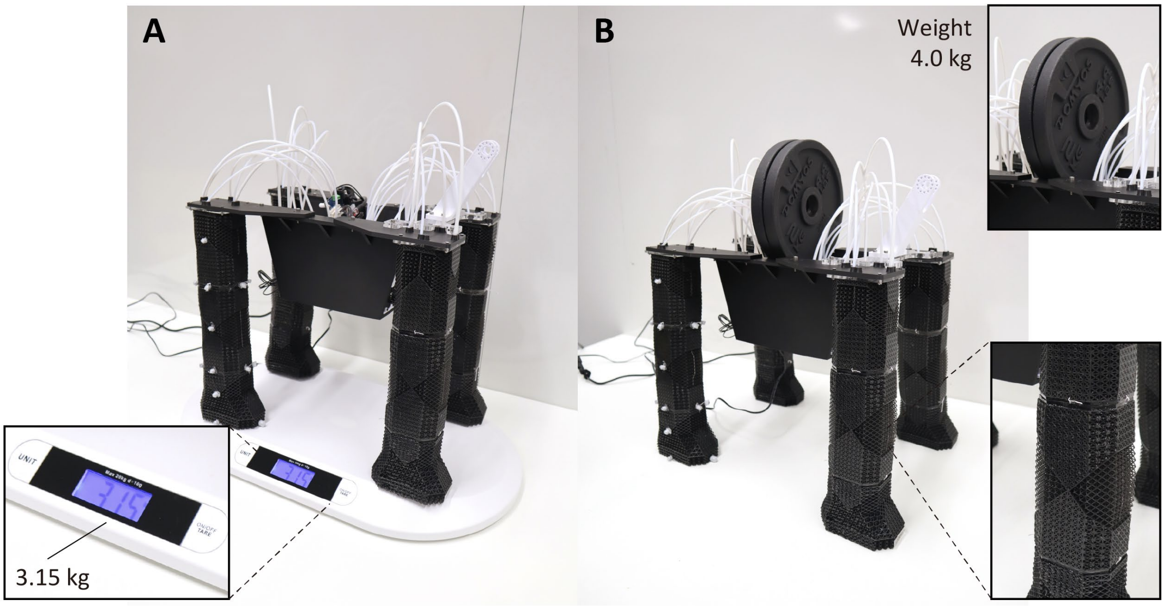

Fig. S18. Load-bearing capacity of the elephant robot. (**A**) The weight of the robot's lower limbs is 3.15 kg. (**B**) Robot under extra 4 kg of weight.

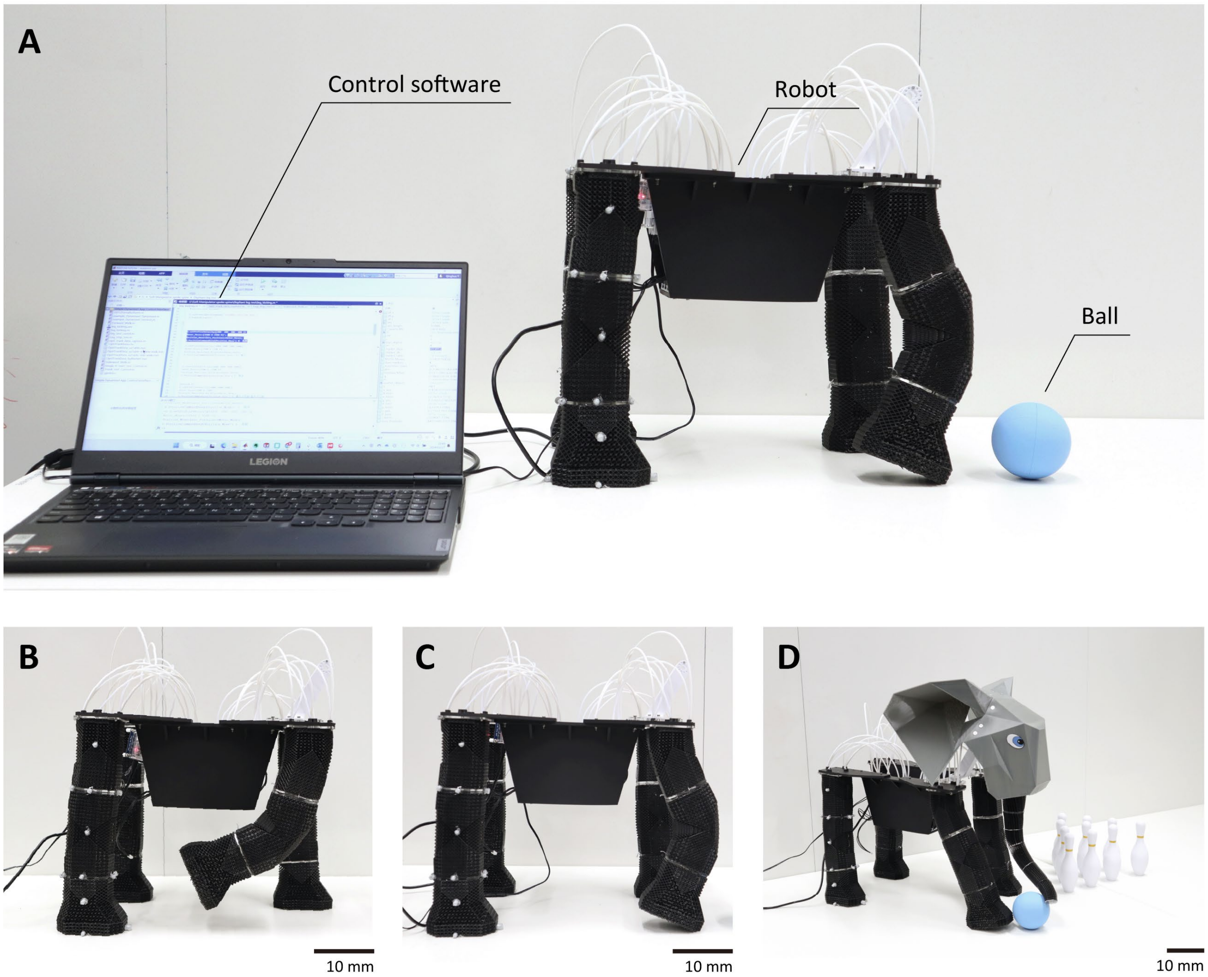

Fig. S19. Elephant leg motion control. (**A**) Set-up to control the elephant legs with Matlab software. (**B**) The elephant lifting its right front leg. (**C**) The elephant standing on tiptoe with its right front leg. (**D**) The elephant playing bowling ball.

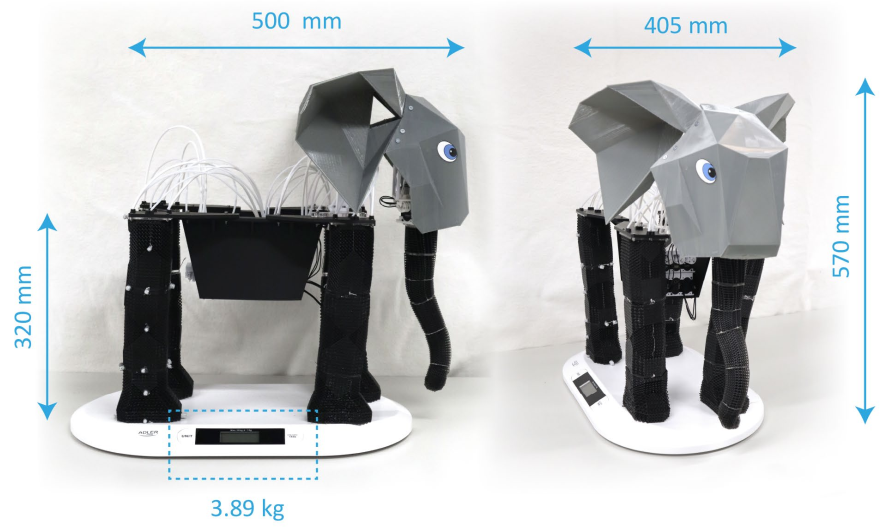

Fig. S20. The dimensions and weight of the elephant robot.

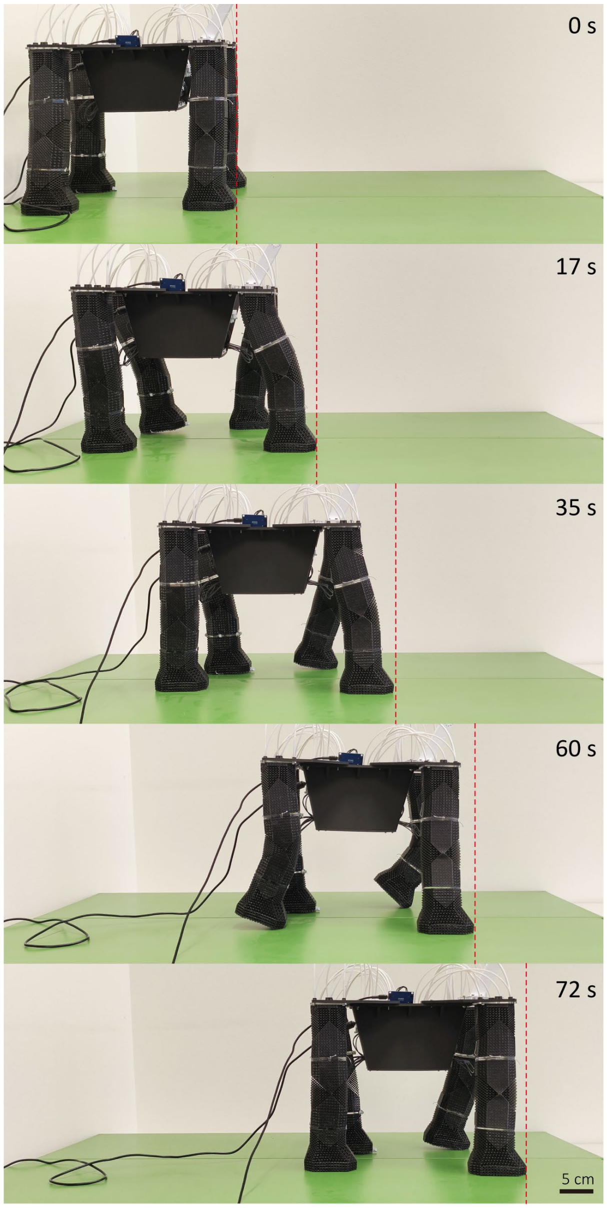

Fig. S21. The sequence photos of robot walking.

Supplementary Tables

Table S1. The number of lattice combinations.

Combination	Superposition	Number
BCC / XCube	1 cell	2
TR	1 cell	5(→∞)
TR + DSP	1 cell	13
DSP + TSP	1 cell	28
TR + TSP	1 cell	36
TR + DSP + TSP	1 cell	100
TR + DSP + TSP	2 cells	4,950
TR + DSP + TSP	3 cells	161,700
TR + DSP + TSP	4 cells	3,921,225
TR + DSP + TSP	5 cells	75,287,520

Table S2. 3D printing parameters of the soft lattice foam.

Layer height (mm)	0.1	Light-off delay (s)	8
Bottom layer Count	4	Lifting distance (mm)	13
Exposure time (s)	5	Lifting speed (mm/min)	90
Bottom exposure time (s)	55		

Supplementary Movies

Movie S1. Trunk movements

Movie S2. Trunk with dynamic grasping capability

Movie S3. Leg gaits

Movie S4. Elephant robot interacting with environment dynamically

Movie S5. Elephant robot walking capability
